# Species boundaries in plant pathogenic fungi: a *Colletotrichum* case study

**DOI:** 10.1186/s12862-016-0649-5

**Published:** 2016-04-14

**Authors:** Fang Liu, Mei Wang, Ulrike Damm, Pedro W. Crous, Lei Cai

**Affiliations:** State Key Laboratory of Mycology, Institute of Microbiology, Chinese Academy of Sciences, Beijing, 100101 China; Microbiology, Department of Biology, Utrecht University, Padualaan 8, 3584 CH Utrecht, The Netherlands; Senckenberg Museum of Natural History Görlitz, PF 300 154, 02806 Görlitz, Germany; CBS-KNAW Fungal Biodiversity Centre, Uppsalalaan 8, 3584 CT Utrecht, The Netherlands; Department of Microbiology and Plant Pathology, Forestry and Agricultural Biotechnology Institute, University of Pretoria, Pretoria, 0002 South Africa

**Keywords:** Coalescent, GCPSR, Mating test, Phylogeny, Species delimitation

## Abstract

**Background:**

Accurate delimitation of plant pathogenic fungi is critical for the establishment of quarantine regulations, screening for genetic resistance to plant pathogens, and the study of ecosystem function. Concatenation analysis of multi-locus DNA sequence data represents a powerful and commonly used approach to recognizing evolutionary independent lineages in fungi. It is however possible to mask the discordance between individual gene trees, thus the speciation events might be erroneously estimated if one simply recognizes well supported clades as distinct species without implementing a careful examination of species boundary. To investigate this phenomenon, we studied *Colletotrichum siamense* s. lat., which is a cosmopolitan pathogen causing serious diseases on many economically important plant hosts. Presently there are significant disagreements among mycologists as to what constitutes a species in *C. siamense* s. lat., with the number of accepted species ranging from one to seven.

**Results:**

In this study, multiple approaches were used to test the null hypothesis “*C. siamense* is a species complex”, using a global strain collection. Results of molecular analyses based on the Genealogical Concordance Phylogenetic Species Recognition (GCPSR) and coalescent methods (e.g. Generalized Mixed Yule-coalescent and Poisson Tree Processes) do not support the recognition of any independent evolutionary lineages within *C. siamense* s. lat. as distinct species, thus rejecting the null hypothesis. This conclusion is reinforced by the recognition of genetic recombination, cross fertility, and the comparison of ecological and morphological characters. Our results indicate that reproductive isolation, geographic and host plant barriers to gene flow are absent in *C. siamense* s. lat.

**Conclusions:**

This discovery emphasized the importance of a polyphasic approach when describing novel species in morphologically conserved genera of plant pathogenic fungi.

**Electronic supplementary material:**

The online version of this article (doi:10.1186/s12862-016-0649-5) contains supplementary material, which is available to authorized users.

## Background

Species are fundamental units for studies in biodiversity, ecology, evolutionary biology, and bio-conservation. A species consists of a population of clones, and the individuals of which can reproduce. Inaccurate delimitation of species may lead to errors in analyses that use species as units (e.g., phylogenetic community structure analyses), and incorrect identification may lead to economic losses in the production, import and export of agricultural and forestry produce, and complications in disease prevention and control [1]. Since the early 90’s mycologists have routinely employed DNA sequence data for the calculation of gene trees and species delimitation. The Genealogical Concordance Phylogenetic Species Recognition (GCPSR) [2] has proven to be a good tool for species delimitation in fungi [3–5], the strength of which lies in its comparison of more than one gene genealogy. According to the GCPSR criteria, conflict among gene genealogies is likely to be due to recombination among individuals within a species, and the incongruence nodes are identified as the point of genetic isolation and species limits. The GCPSR is especially practical for delimiting species in morphologically reduced fungi. Nevertheless, species boundaries of closely related taxa, in the initial stages of divergence, can be difficult to ascertain using multi-locus phylogenetic methods because genes can differ substantially in their evolutionary histories [6]. Processes such as incomplete lineage sorting, recombination, horizontal gene transfer and population structure could cause discordances between gene trees and species trees, masking true evolutionary relationships among closely related taxa [7]. Furthermore, the common approach of concatenating sequence data from multiple loci can also lead to poor species discrimination [8].

Alternatively, coalescent-based species delimitation methods, such as General Mixed Yule Coalescent (GMYC), Poisson Tree Processes (PTP) and Bayesian Phylogenetics and Phylogeography (BPP), could incorporate the process of lineage sorting and the presence of incongruent genomic regions into phylogenetic estimation procedures [9]. This is an important distinction from GCPSR because most alleles are not expected to be reciprocal monophyletic among lineages across most of the genome, particularly at the timescale of recent speciation [10]. Estimating the species tree and species delimitation using coalescent methods for closely related taxa have proven very useful and have been used for a range of animal and plant taxa [11–19]. These methods have otherwise not been much used in fungi, especially in studies of plant pathogenic fungi [20].

*Colletotrichum siamense* [21], a member of the *C. gloeosporioides* complex, is a cosmopolitan and host diverse species on fruits, leaves and seeds [22–25]. From 2009 to 2014, seven species with close phylogenetic affinities to *C. siamense* have been described, i.e. *C. communis* [26], *C. dianesei* [27], *C. endomangiferae* [28], *C. hymenocallidis* [29], *C. jasmini-sambac* [30], *C. melanocaulon* [31] and *C. murrayae* [32]. They were regarded as species within *C. siamense* s. lat. in some publications [23, 28, 33]. In a recent study of the *C. gloeosporioides* species complex [22], *C. hymenocallidis* and *C. jasmini-sambac* were synonymized with *C. siamense* s. str. based on a five-locus phylogenetic analysis (*ACT*, *CAL*, *CHS1*, *GAPDH*, *ITS*). Sharma et al. [26], however, resurrected *C. hymenocallidis* and *C. jasmini-sambac* and accepted seven species including one additional new species in the *C. siamense* species complex. These developments have led to significant disagreements regarding the status of *C. siamense* s. lat, either as single species or species complex.

Most species in the “*C. siamense* species complex” were proposed and analyzed based on the concatenation of different loci without strictly complying with GCPSR. Among them, *C. dianesei*, *C.jasmini-sambac*, *C. hymenocallidis* and *C. siamense* were proposed based on six combined loci (*ACT*, *CAL*, *GAPDH*, *GS*/*CHS1*, *ITS*, *TUB2*), *C. endomangiferae* based on a single locus (*Apn2/MAT IGS = ApMat*) and six combined loci (*ACT*, *CAL*, *GAPDH*, *CHS1*, *ITS*, *TUB2*), *C. melanocaulon* based on three loci (*ApMat*, *ITS*, *TUB2*), and *C. murrayae* based on six combined loci (*ACT*, *CAL*, *GAPDH*, *GS*, *ITS*, *TUB2*). Hitherto, *ApMat* has been shown to be the most phylogenetically informative locus compared to other commonly used loci (*Apn25L*, *MAT5L*, *MAT1-2-1*, *ITS*, *TUB2*, *GS*) in the *C. gloeosporioides* species complex [34]. Researchers have thus tried to resolve species delimitation by solely employing ApMat analysis [26, 28, 33]. *Colletotrichum communis* was proposed as a novel species in the “*C. siamense* species complex” based on an *ApMat* analysis, even though there was incongruence with the multi-locus tree [26]. Species recognition based on a single locus can result in species identification that does not reflect true evolutionary relationships, because of the existence of incongruent loci, and because the resulting clades could display variability above or below species level.

The objective of this study was thus to test the null hypothesis that *C. siamense* s. lat. is a species complex by implementing a polyphasic approach that includes comparison of morphological characteristics, both single- and multi-locus phylogenetic analyses, pairwise homoplasy index test, mating compatibility test, and coalescent-based species delimitation methods comprising GMYC, PTP and BPP.

## Results

### Phylogenetic analyses

**Fig. 1:**
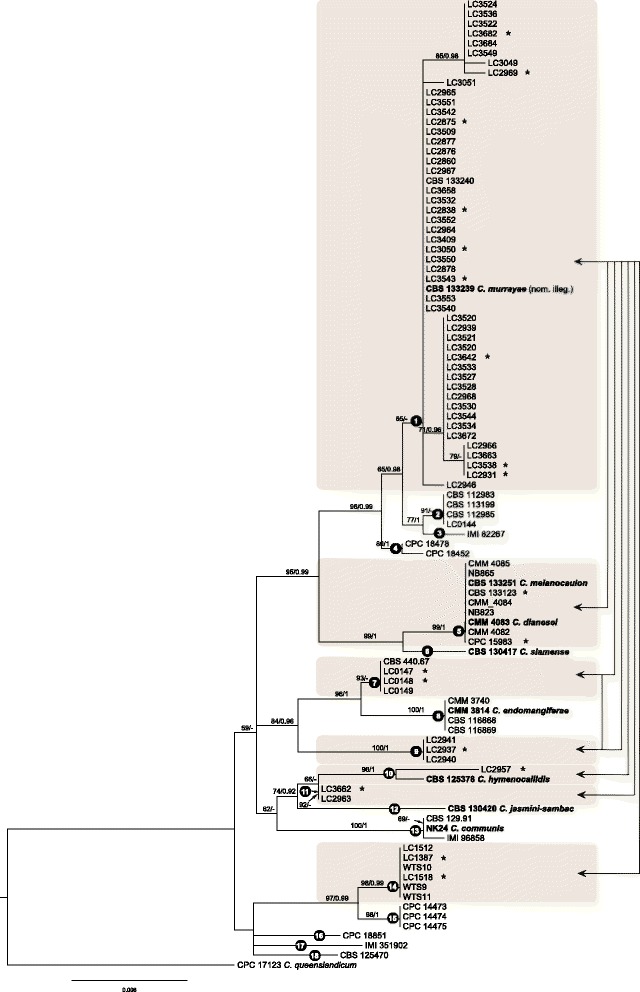
Phylogenetic tree of *C. siamense* s. lat. calculated with a maximum likelihood analysis of ApMat sequences by running RAxML v.7.0.3. The RAxML bootstrap support values (ML, > 50 %) and Bayesian posterior probabilities (PP, > 0.95) are displayed at the nodes (ML/PP). Eighteen clades (clade 1 to 18) are designated in the tree. Ex-type isolates are emphasized in bold. Stars indicate isolates used for mating test, and colored blocks pointed by double-headed arrows link cross fertile clades (for details see Additional file 8: Table S2)

Phylogenetic analyses of 98 strains of *C. siamense* s. lat. were performed on single locus and concatenated datasets. The full sequence length, alignment length with gaps, number of informative characters and substitution model of each locus are stated in Table 1. The topologies of the ML and BI trees confirmed each other, and only the ML trees of each single locus, five combined loci (*CAL*, *GAPDH*, *GS*, *ITS*, *TUB2*) and eight combined loci were shown in Fig. 1 & Additional file 1: Figure S1. A total of 18 potential “species”, i.e. clade 1 to clade 18, were temporarily designated based on the bootstrap values/posterior probabilities and branch lengths in the *ApMat* phylogram (Fig. 1), combining with the treatment of these corresponding clades and “species” in a previous publication [26], as well as the geographical distribution and hosts of the strains in Fig. 1. Although the bootstrap value of clade 1 is relatively low, the related clades 2–4 were all supported with high bootstrap values or posterior probabilities. In addition, all strains in group1 were from China, while most of the strains in clade 2 were from Africa, and clades 3 and 4 were from Brazil. This designation is consistent with the classification system of *C. siamense* s. lat. in the recent publication of Sharma et al. [26]. Subsequently, congruencies/discordances of phylogenies of the single loci and different combinations of loci compared to the *ApMat* phylogeny are plotted in a heat map (Table 1). In Table 1, clades were ordered according to the discordant levels compared to the *ApMat* phylogeny. All single locus phylogenies were incongruent with the *ApMat* phylogeny (see red color in Table 1). Even the topologies of the flanking regions of *ApMat*, *Apn25L* and *MAT1-2-1*, were slightly different from the *ApMat* phylogeny, which were reflected by clade 1 and clade 7 on *Apn25L* gene tree, and clade 1 on the *MAT1-2-1* gene tree (Fig. 1 & Additional file 1: Figure S1).

**Table 1:**
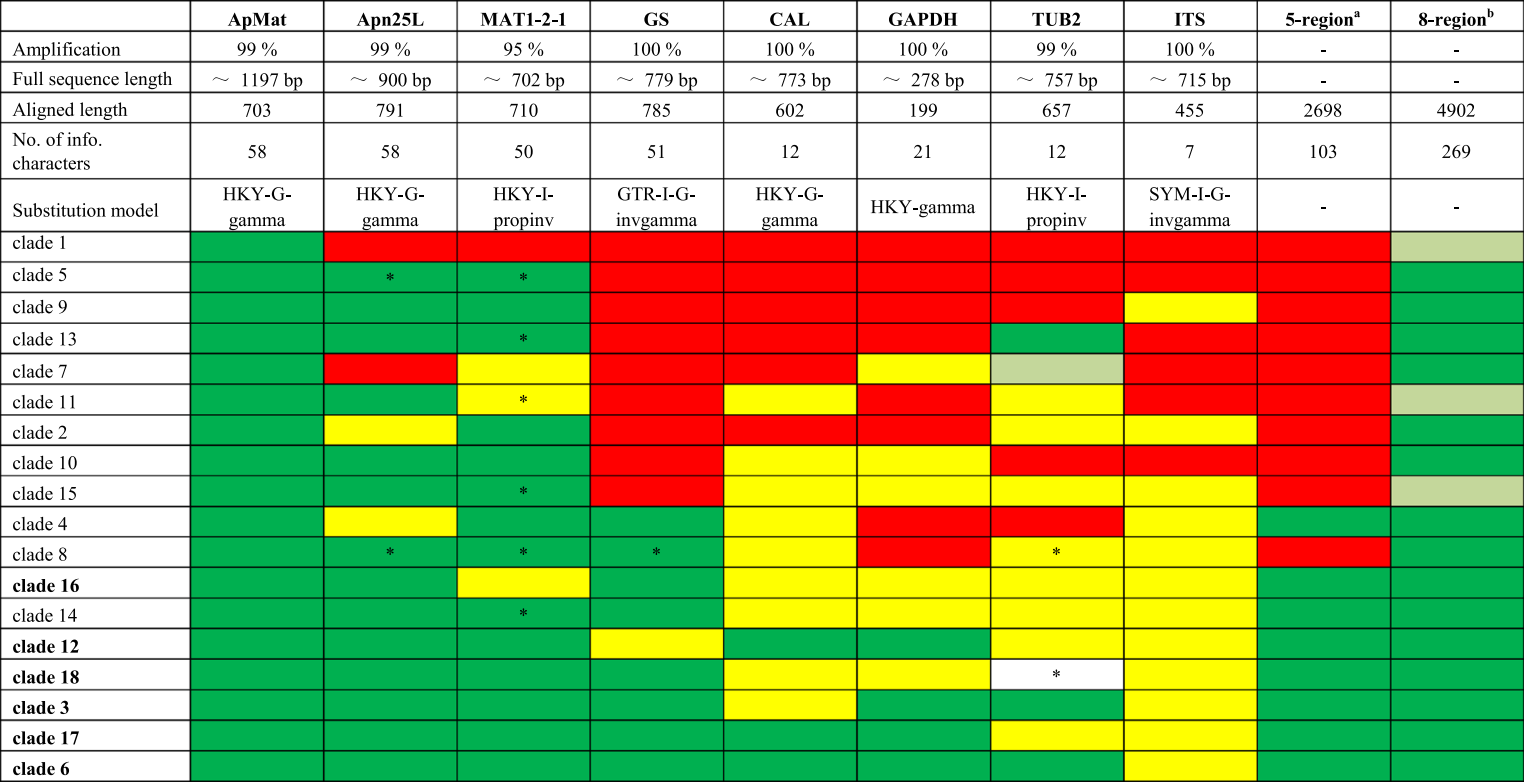
Summary of locus and phylogenetic results as well as heat map of congruencies/conflicts of phylogenies compared to ApMat phylogeny

Note: a: CAL, GAPDH, GS, ITS, TUB2. b: CAL, GAPDH, GS, ITS, TUB2, ApMat, Apn25L, MAT1-2-1. Green color: congruent topology with ApMat tree; olive color: isolates of that clade are polyphasic, but distinguishable from other clades; yellow color: isolates of that clade grouped together, but indistinguishable from other clades;red color: isolates of that clade are polyphasic, and indistinguishable from other clades. *: dataset is incomplete. Clades composed of single isolate are in bold

**Fig. 2:**
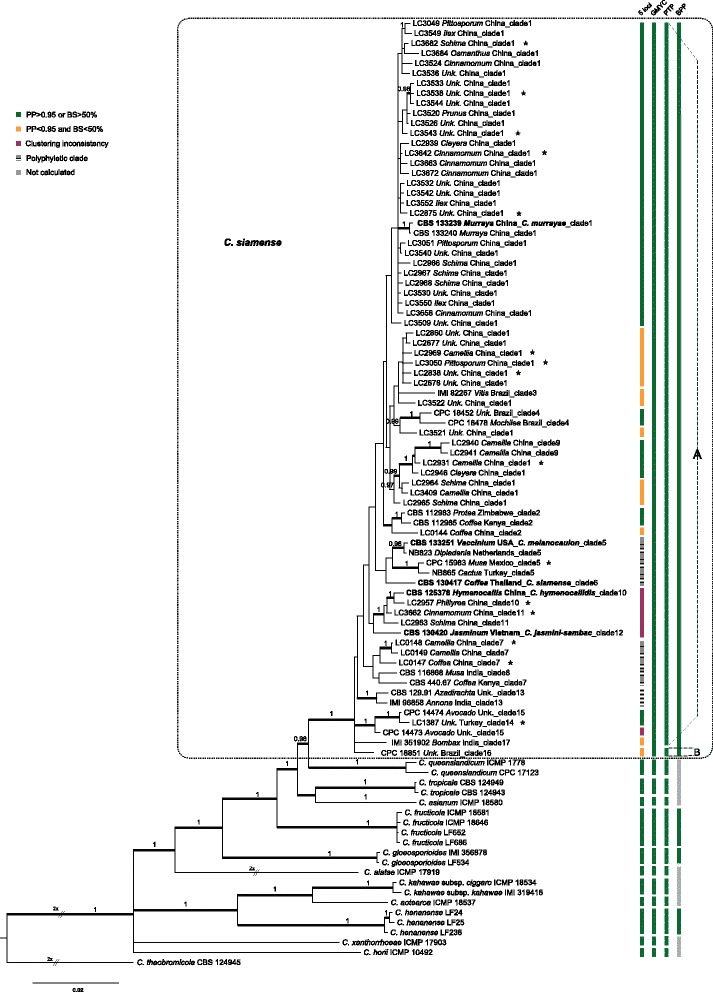
Phylogenetic relationships and species boundaries of *C. siamense* s. lat. and related species. Fifty percent majority rule consensus tree from a Bayesian analysis based on a six-locus combined dataset (ApMat, CAL, GAPDH, GS, ITS, TUB2). Posterior probabilities (PP, > 0.95) are displayed at the nodes. Thickened branches indicate branches also present in the ML tree with > 50 % bootstrap support values. Bars in the first column at the right present the results of the phylogenetic analysis based on five-locus (CAL, GAPDH, GS, ITS, TUB2) alignment, respectively. The other three columns present the results of three coalescent-based species delimitation methods (GMYC, PTP, BPP). “A” and “B” represent the two potential species inferred from PTP analysis. Ex-type cultures are emphasized in bold. Stars indicate isolates included in the mating test

Seventy-four haplotypes of *C. siamense* s. lat. and 21 haplotypes of well-delimitated species in the *C. gloeosporioides* complex were included in the further phylogenetic analyses*.* The dataset included 748 characters with alignment gaps for *ApMat*, 613 for *CAL*, 221 for *GAPDH*, 798 for *GS*, 458 for *ITS*, and 635 for *TUB2*. For the Bayesian inference, a GTR + I + G model with inverse gamma-distributed rate was selected for *ApMat*, a HKY + G model with gamma-distributed rates for *CAL*, a GTR + G model with gamma-distributed rates for *GAPDH* and *ITS*, HKY + I model with propinv-distributed rate for *GS* and a SYM + G model with gamma-distributed rate for *TUB2*. ML trees confirmed the tree topologies of the BI trees. Results of the phylogenetic analyses are presented in Fig. 2. For the single locus analyses, we only showed the *ApMat* tree to compare the topology with that of the six-locus tree. Although a few subclades within *C. siamense* s. lat. were strongly supported on the six-locus tree, e.g. clades with ex-type of *C. melanocaulon* and *C. hymenocallidis* respectively, the deeper nodes were poorly supported (Fig. 2). In addition, some strongly supported subclades in *C. siamense* s. lat. in the six-locus tree were polyphyletic or poorly supported in the *ApMat* and five-locus trees (Fig. 2), and vice versa. In contrast, the well-delimitated reference species were well supported either in single locus or in concatenated gene trees.

Significant recombination was detected among the strains of *C. siamense* s. lat. in many different clades when applying PHI tests with the GCPSR model (Additional file 2: Table S1), which indicated that there was no reproductive isolation within the group. Subsequently, single ML trees (*ApMat*, *CAL*, *GAPDH*, *GS*, *ITS*, *TUB2*) of *C. siamense* s. lat. and related species were combined into a phylogenetic network (Additional file 3: Figure S2). Based on the relative distance of species and structure of the phylogenetic network, all tested strains in *C. siamense* s. lat. should be assigned to one single species (Additional file 3: Figure S2). Therefore, the null hypothesis that *C. siamense* s. lat. is a species complex was rejected by implementing GCPSR.

### Species delimitation based on coalescent methods

Regarding the GMYC analyses, both single-threshold and multiple-threshold GMYC models resulted in significantly better fit to the ultrametric tree than the null model and recovered *C. siamense* s. lat. as one entity (i.e., potential species) (Fig. 2, Additional file 4: Figure S3 & Additional file 5: Figure S4). For the PTP analysis, two potential species were inferred from *C. siamense* s. lat., designated as A and B (Fig. 2), based on the best-fit ML tree and BI majority-rule consensus topology (Additional file 6: Figure S5). Compared with the results of the GMYC analyses, the only difference was that a single strain, CPC 18851, clustered apart from *C. siamense* s. lat.

In order to test the validity of the hypothesized species inferred from PTP, BPP analyses were performed. The dataset was composed of strains of the two potential species, A and B, that resulted from the PTP analysis and three reference species, *C. fructicola*, *C. gloeosporioides* and *C. henanense*. Both analyses with a small ancestral population size (G*θ*s (2, 1000)) supported four species, i.e., A&B (A and B as one), *C. fructicola*, *C. gloeosporioides* and *C. henanense*, with high posterior probabilities (Table 2), and the delimited species A&B was strongly supported (pp = 1.00 or 0.94). Analyses with a large ancestral population size (G*θ*s (1, 10)) gave unconvincing results because the posterior probabilities were very low (<0.90, Table 2) (Leache and Fujita [35]; Yang and Rannala [36]), in other words A and B were not supported as two distinct species. Therefore, the prior with small ancestral population size and shallow divergence is superior, which recovered the entire *C. siamense* s. lat. as one species by performing BPP analyses. Overall the coalescent-based species delimitation methods gave mostly congruent results that rejected the null hypothesis.

**Table 2:** Results from BP&P analyses for *C. siamense* s. lat. assuming a 2-species model

Priors	Posterior probability	pp for delimited species					
		A	B	A&B	*C. fructicola*	*C. gloeosporioides*	*C. henanense*
G*θ*s (1, 10) ~ Gτ0 (1, 10)	P[5] = 0.72	0.72	0.72	0.27	1.00	1.00	1.00
G*θ*s (1, 10) ~ Gτ0 (2, 1000)	P[5] = 0.80	0.80	0.80	0.20	1.00	1.00	1.00
G*θ*s (2, 1000) ~ Gτ0 (1, 10)	P[4] = 1.00	-	-	1.00	1.00	1.00	1.00
G*θ*s (2, 1000) ~ Gτ0 (2, 1000)	P[4] = 0.94	0.05	0.05	0.94	1.00	1.00	1.00

### Mating test

Mature perithecia and oozing ascospores were observed on pine needles approximately 1–2 months after inoculation (Additional file 7: Figure S6). Cross fertility was observed in 43 of the 106 combinations tested, which corresponded to 41 % (Additional file 8: Table S2). Strains belonging to different clades of the phylogenetic trees (Figs. 1 & 2) could mate and produce perithecia and abundant viable ascospores (Additional file 7: Figure S6), which indicated that reproductive isolation was not present. Nevertheless, these tested strains could not be separated into two distinct incompatibility groups. For example, LC2838 and LC2931 were cross-fertile, but both of which could cross with strains LC3642, LC3682, LC0148, LC2937 and LC3662.

### Morphological analysis

Based on the morphological observations, 40 sporulating strains of *C. siamense* s. lat. were selected for the hierarchical clustering analysis. A dendrogram was produced by the Ward’s method based on the data of conidial length and width, which could be divided into three distinct large clusters (Additional file 9: Figure S7). However, the dendrogram based on conidial measurements did not correspond to any of the molecular phylograms of *C. siamense* s. lat.

#### Taxonomy

The present study incorporated phylogenetic analyses based on GCPSR criteria and coalescent species tree estimation, cross mating test and morphological comparisons to delimit species within *C. siamense* s. str. and related taxa. *Colletotrichum communis*, *C. dianesei*, *C. endomangiferae*, *C. hymenocallidis*, *C. jasmini-sambac*, *C. murrayae* and *C. siamense* are confirmed to be conspecific, which constitutes a single species infecting various host plants worldwide.

- *Colletotrichum siamense* Prihast., L. Cai & K.D. Hyde, Fungal Diversity 39: 98 (2009)
- *= Colletotrichum communis* G. Sharma, A.K. Pinnaka & B.D. Shenoy, Fungal Diversity 71: 256 (2015)
- *= Colletotrichum dianesei* N.B. Lima, M.P.S. Câmara & S.J. Michereff, Fungal Diversity 61: 83 (2013)
- *= Colletotrichum endomangiferae* W.A.S. Vieira, M.P.S. Camara & S.J. Michereff, Fungal Diversity 67: 192 (2014)
- *= Colletotrichum hymenocallidis* Yan L. Yang, Zuo Y. Liu, K.D. Hyde & L. Cai, Fungal Diversity 39: 138 (2009)
- *= Colletotrichum jasmini-sambac* Wikee, K.D. Hyde, L. Cai & McKenzie, Fungal Diversity 46(1): 174 (2011)
- *= Colletotrichum melanocaulon* V.P. Doyle, P.V. Oudem. & S.A. Rehner, PLoS ONE 8: e62394 (2013)
- *= Colletotrichum murrayae* Li J. Peng & K.D. Hyde, Cryptogamie, Mycologie 33: 278 (2012) (nom. illegit.)

Description and illustrations –– See Prihastuti et al. [21], Yang et al. [29], Wikee et al. [30], Doyle et al. [31], Peng et al. [32], Lima et al. [27], Vieira et al. [28], Liu et al. [24], Sharma et al. [26].

## Discussion

Accurate species identification of the causal organism of plant disease is crucial for disease control and prevention. Although the criteria used to delimit and identify species of plant pathogenic fungi have changed over time, they could be classified as morphological, biological, ecological and phylogenetic species recognition [2, 37, 38]. The importance of recognizing cryptic species of plant pathogenic fungi has been widely underscored, and such studies have increased exponentially over the past decades [39–42]. It has been largely fuelled by the increasing availability of DNA sequences, with the aid of phylogenetic analyses based on one or multi-locus sequence data. Most researchers, however, did not carefully examine the species boundaries, but simply recognize distinct clades in either single- or multi-locus trees as species [6]. The recognition of distinct clades in gene trees as species is likely to be misleading in understanding the evolutionary history of taxa. Even different populations may separate into distinct clades when using tree reconstruction methods, since this is the dominant signal in the data. However, it might not be the sole signal that could be used for species recognition. In other words, a gene tree is not necessarily corresponding to the species tree. For example, high intraspecific variation in *ITS* sequences was detected within the *Ceratocystis fimbriata* complex, and species previously described on that basis were revealed to be *ITS* haplotypes [43, 44].

### Genealogical concordance phylogenetic species recognition (GCPSR)

Supported nodes in a single gene tree might be in conflict with those in the concatenated multi-locus tree, as well as in the other single gene trees. Gatesy and Baker [45] noted that the combination of multiple loci, which separately do not support a clade, often reveals emergent support for or conflict within that clade. In the case of *C. siamense*, most clades received strong support in the 8-locus tree, but were manifested as polyphyletic or poorly supported in the single locus and 5-locus trees (Additional file 1: Figure S1, Table 1), because the shorter alignments used for single and 5-locus trees provided less power to resolve all splits.

According to the GCPSR criteria, the lack of genealogical congruence among gene trees is a signal that the sampled diversity is below species level [2]. In contrast, concordance between gene trees can provide strong evidence for the distinct and congruent clades to represent reproductively isolated lineages. In the phylogenetic analyses of *C. siamense* s. lat., conflicts were discovered between any pair of single locus phylograms, or even concatenated gene trees (Additional file 1: Figure S1 & Additional file 10: Figure S8, Table 1). Therefore, the null hypothesis was rejected by implementing GCPSR criteria. Besides, the topology of the *ApMat* phylogram proved to be almost congruent with that of the 8-locus phylogram (Fig. 1, Additional file 1: Figure S1). It is possible that mating-related genes evolve at a faster rate and have a higher sequence variability, which therefore dominates the topology of the multi-locus phylogram. In addition, single-locus data inferred the evolutionary history of relationships of a single gene but not that of the organisms [46, 47]. For example, in the *Rhizoplaca melanophthalma* species complex, the ITS topology differed greatly from the coalescent-based species tree estimated from multi-locus sequence data [47]. Therefore, the use of multi-locus sequence data is essential to establish robust species boundaries [48].

To further apply the GCPSR criteria to the *C. siamense* s. lat. dataset, the 18 clades recognized in the *ApMat* tree were tested for genetic exchange to indicate their evolutionary independence. The resulting pairwise homoplasy index test revealed significant genetic recombination among almost half of the paired clades. Strains in seven clades (i.e., clade 1, 2, 3, 5, 7, 8, 9) showed genetic recombination with strains in more than 10 of the other clades, which supports the alternative hypothesis that *C. siamense* s. lat. is not a species complex. It is noteworthy that strains from *Persea americana* in clade 14 only show recombination with strains in clade 8 (host: *Mangifera* and *Musa*) and clade 15 (Host: *Persea americana*), which supports most of the phylogenetic analyses that strains of clades 14 and 15 always clustered together. The recombination between clade 14 and the other clades was probably minimized over time due to the adaptive divergence and ecological allopatry of strains occurring on *Persea americana*.

### Species estimation using coalescent methods

Although the concatenation of multi-locus DNA sequences is powerful and convenient in calculating phylogenetic trees, these trees might not be congruent with the species trees [13, 49, 50]. Therefore, researchers have recently called for methods based on the coalescent theory [7, 13, 15], which can make quantitative predictions about probabilities of gene trees, and serve as a baseline for investigating causes of gene tree discordance, e.g. incomplete lineage sorting, horizontal gene transfer, gene duplication and loss, hybridization, and recombination [7]. These methods could avoid arbitrary cut-offs [51] and over-supporting poorly resolved clades [52]. Belfiore et al. estimated species trees using concatenation and BEST (Bayesian Estimation of Species Tree, a coalescent method) methods for pocket gophers *Thomomys*, and found that species were over-estimated using the concatenated analysis, whereas fewer were supported in the phylogeny estimated using BEST [52]. Their result is similar to that of our study on *C. siamense* s. lat. In the present study, many clades within *C. siamense* s. lat. in the concatenated gene trees were well supported and some of them had been described as species. However, the results by implementing coalescent methods were entirely contrary. GMYC analysis inferred *C. siamense* s. lat. as one species, while PTP analysis separated *C. siamense* s. lat. into two entities (i.e., “species”), A and B. However, the separation of A and B was not supported by the BPP analysis, even though it had good power in the recognition of distinct species in the presence of small amounts of gene flow [53]. In other words, over-estimated species in *C. siamense* s. lat. obtained in concatenated multi-locus analyses were not supported by coalescent-based analyses.

### Biological, morphological and ecological species recognition

Studies of cross fertility, morphological and geographical characteristics are also used in species delimitation. The Biological Species Concept defines species in terms of interbreeding. Nevertheless, mating behavior in fungal species depends not only on the compatibility, but also on environmental factors such as habitat/medium, illumination, pH, humidity, and temperature and other factors [54]. Thus fungal cross fertility or sterility was not theoretically sufficient to reject or approve the null hypothesis in the present study. However, cross fertility among strains in different clades did prove that reproductive isolation was not formed and supported the conclusion of GCPSR and coalescent analyses, i.e., *C. siamense* is one species.

The Morphological Species Recognition emphasizes morphological divergence and is widely applied to differentiate organisms [55]. However, with the application of molecular methods in fungal taxonomy in recent years, phylogenetic diversity has been discovered within morphologically defined species. The genus *Colletotrichum* is a typical example [22, 56]. In our study, the morphological distinctiveness or indistinctiveness was neither sufficient to reject nor to prove the null hypothesis. Regarding the dendrogram of conidial length and width, three groups were differentiated. However, they were not consistent with clades of any of the molecular phylograms of *C. siamense* s. lat. calculated in this study. Therefore, even though the result of the morphological comparison is insufficient to reject the null hypothesis, it was clearly prone to support the one species hypothesis and apparently just reflects the variability in conidia size within *C. siamense*.

As to ecological species recognition [57], a species is a lineage or a closely related set of lineages that occupies an adaptive zone minimally different from that of any other lineage in its range, which is however, not always obvious and easy to observe in nature. Distinct lineages recognized in the phylogenetic tree can be used as guide for finding diagnostic ecological differences among clades. In our study, none of the well-supported clades is restricted to a specific locality, which indicates the absence of a geographic barrier in gene flow in nature. In addition, no host-specific clade is revealed, and strains from the frequently sampled hosts (e.g. *Camellia*, *Schima*, *Coffea*) appeared in different clades throughout the *C. siamense* tree (Fig. 2 & Additional file 1: Figure S1). In other words the null hypothesis was rejected according to ecological species criteria.

### Importance of a large sampling size in species delimitation

The phylogenetic species concept is based on the assumption that the fixation of a particular character state in a population is diagnostic of a long history of reproductive isolation [58]. In practice, species recognition is usually based on the characters of a small group of individuals rather than that of entire populations of a particular species. Thus unfortunately, individuals of a small sample size sharing one unique character can often be easily drawn out from populations of a particular species, which is actually polymorphic. In other words, one or only a few individuals often fail to represent the species as a whole, especially for those with widespread distributions [58–60]. If two divergent populations present certain morphological or genetic distinctions, new species might be mistakenly described. In Gao et al. [61], it was demonstrated that adding a number of new strains into a group containing two originally well supported sister clades (recognized as distinct species in previous studies) may completely erase the distinctiveness of the two clades. The “species” within *C. siamense* s. lat. demonstrate a similar situation. Many recognized species were proposed based on few strains, i.e., *C. siamense* s. str. and *C. jasmini-sambac* were each based on three strains [21, 30], while *C. endomangiferae*, *C. hymenocallidis* and *C. melanocaulon* were respectively based on two strains [28, 29, 31]. This appears to be one of the main reasons that led to ambiguous species boundaries. For example, although sister clades of *C. melanocaulon* and *C. siamense* s. str. received strong support values in Doyle et al. [31], their distinctiveness were not supported when adding more strains in this group in the present study. Therefore, obtaining a sufficient number of strains from diverse origins is crucial for delimiting species or introducing a novel species in *Colletotrichum* and similar genera of plant pathogenic fungi with a conserved morphology.

Incongruence between gene trees and species trees is commonly detected in multi-locus analyses, and the process of incomplete lineage sorting is a potential source of discordance [13]. Incomplete lineage sorting occurs when recently diverged lineages retain ancestral polymorphism because they have not had sufficient time to achieve reciprocal monophyly [10]. In general, the lack of complete lineage sorting would not be revealed without using multiple individuals per taxon [62]. To date, a large number of cryptic animal and plant species have been discovered using coalescent approaches that explicitly model the discordance between gene trees and species trees that resulted from the incomplete lineage sorting [6, 12, 13, 15]. However, these approaches are seldom applied in fungi, especially in parasitic fungi [20]. In the present study, 98 strains of *C. siamense* s. lat. from 14 countries and more than 29 hosts were demonstrated to represent a single species using several coalescent methods.

### The importance of a polyphasic approach

Although various species recognition criteria have been developed to delimit species, using sole or a few criteria might minimize the discovery of cryptic species or overestimate species numbers. For example, based on morphological characteristics with little emphasis on pathological features, accepted species of *Colletotrichum* were reduced from around 750 to 11 [63]. However, three of the 11 species have subsequently been demonstrated to represent a species complex containing many cryptic species based on multiple approaches [40]. Underestimation of cryptic species has been manifested in many other plant pathogenic fungal genera using molecular data analyses, i.e. *Althernaria* [42, 64], *Bipolaris* [65], *Ceratocystis* [66], *Diaporthe* [41], *Phoma* [67], *Pyricularia* [68], and *Septoria* [38].

In recent years, polyphasic approaches have been strengthened to reflect the natural classification of species within many important fungal genera, i.e. *Cladobotryum* [69], *Colletotrichum* [37], *Phoma* and related species [70], and genera in Teratosphaeriaceae [71]. This approach commonly incorporates morphological, physiological and phylogenetic analyses, pathogenicity tests, and metabolomics, but seldomly employ coalescent species tree estimation, which was demonstrated to be particularly objective and useful in species delimitation for closely related taxa of animals and plants [14–19]. Based on our findings it is recommended that mycologists in future employ a polyphasic approach to delineate species in morphologically conserved genera, where simply single-locus or concatenated phylogenetic analyses and small sample size could lead to an inflation of species numbers, which in turn could have serious implications for trade, disease control and prevention.

## Conclusions

Results of molecular analyses based on GCPSR and coalescent methods of GMYC, PTP and BPP proved that *C. siamense* s. lat. is single species rather than a species complex [26]. Further analyses, i.e. PHI test, cross fertility and the comparison of ecological characters, reinforced that reproductive isolation, geographic and host plant barriers to gene flow among hypothesized “species” in *C. siamense* s. lat. have not formed. This discovery demonstrated that speciation events might be overestimated in fungi if all well-supported clades are accepted as distinct species when using phylogenetic analysis of single-locus or concatenation of multi-locus DNA sequence data on a small sample size. The polyphasic approach in this study provided us a sound scenario for species delimitation and can be applied, in principle, to any fungal species that are morphologically indistinguishable. Furthermore, this study emphasized the importance of a large sampling size in species delimitation.

## Methods

### Strains

Wile-type isolates of a fungus are referred to as strains once characterized. In the present study strains of *C. siamense* s. lat. were selected based on preliminary phylogenetic analyses of *GAPDH* and *ApMat* sequences from the LC culture collection (personal culture collection of Lei Cai housed in the Institute of Microbiology, Chinese Academy of Sciences), the culture collection of the CBS-KNAW Fungal Biodiversity Centre, Utrecht, the Netherlands (CBS), and the CPC culture collection (working collection of Pedro W. Crous, housed at CBS). In total, 98 strains of *C. siamense* s. lat. were analyzed (Additional file 11: Table S3). These strains were from various host plants from 14 countries, including the ex-type cultures of *C. siamense* s. str., *C. hymenocallidis*, *C. jasmini-sambac*, *C. melanocaulon* and *C. murrayae*. Ex-type cultures of other related taxa, i.e. *C. dianesei*, *C. communis* and *C. endomangiferae*, were not available to us, but their sequences and those of related species belonging to the *C. gloeosporioides* complex were downloaded from GenBank (www.ncbi.nlm.nih.gov/genbank).

### DNA extraction, PCR amplification and sequencing

Total genomic DNA was extracted from axenic cultures with a modified CTAB protocol as described in Guo et al. [72]. Eight loci were amplified and sequenced, which are the Apn2-Mat1-2 intergenic spacer and partial mating type Mat1-2 gene (*ApMat*), partial sequences of the Apn2 (*Apn25L*), calmodulin (*CAL*), beta-tubulin (*TUB2*), glutamine synthetase (*GS*) and the mating type (*MAT1-2-1*) genes, an intron of the glyceraldehyde-3-phosphate dehydrogenase (*GAPDH*) gene, and the 5.8S nuclear ribosomal gene with the two flanking transcribed spacers (*ITS*).

PCR primers used in this study are shown in Additional file 12. The PCR with *GS* primers (GSF1 & GSR1, GSF3 & GSR2) used in Stephenson et al. [73] and Weir et al. [22] resulted in non-specific products with some strains. Therefore, new primers (GSLF2, GSLF3 and GSLR1) were designed for *Colletotrichum* based on *GS* sequences generated from GSF1 & GSR1 (Additional file 12: Table S4).

PCR amplification protocols were performed as described by Damm et al. [74], but the denaturing temperatures were adjusted to 52 °C for *ApMat*, *Apn25L*, *CAL*, *GAPDH*, *GS* (GSF1 & GSR1) and *ITS*, 48–62 °C for *MAT1-2-1* and 55 °C for *GS* (GSLF2 or GSLF3 & GSLR1) and *TUB2*. Touchdown PCR programs were used if the amplicons of *GS* and *TUB2* resulted in double bands. Briefly, the annealing temperature started at 62 °C and decreased, in steps of 0.7 °C per cycle, to 54 °C; then another 30 cycles were performed with an annealing temperature of 54 °C. The DNA sequences obtained from forward and reverse primers were used for consensus sequences using MEGA v.5.1 [75]. Subsequent alignments for each gene were generated using MAFFT v.7 [76] and improved where necessary using MEGA v.5.1. Single gene alignments were then concatenated with Mesquite v.2.75 [77]. All novel sequences were deposited in NCBI’s GenBank database, and the alignments in LabArchives (http://www.labarchives.com/).

### Phylogenetic analyses

#### Phylogenetic analyses of *C. siamense* s. lat

Phylogenetic analyses of *C. siamense* s. lat. (Additional file 11: Table S3) were carried out based on single locus (*ApMat*, *Apn25L*, *CAL*, *GAPDH*, *GS*, *ITS*, *MAT1-2-1*, *TUB2*) and concatenated multi-locus datasets. Bayesian inference (BI) and Maximum Likelihood (ML) methods were implemented in this study. Bayesian analyses were performed using MrBayes v.3.2.2 [78] as outlined by Liu et al. [79]. Evolutionary models were estimated in MrModeltest v.2.3 using the Akaike Information Criterion (AIC) for each locus [80] and applied to each gene partition. ML analyses were performed using RAxML v.7.0.3 [81] with 1000 replicates under the GTR-GAMMA model. Subsequently the congruencies/discordances of the resulting phylogenies of the single locus and different combinations of loci were plotted on a heat map.

#### Phylogenetic analyses of *C. siamense* s. lat. and related species

Since there were no *Apn25L* and *MAT1-2-1* sequences available for most of the species in the *C. gloeosporioides* complex, ML and BI analyses of *C. siamense* s. lat. and related species were performed on six single loci (*ApMat*, *CAL*, *GAPDH*, *GS*, *ITS*, *TUB2*) and the respective concatenated multi-locus dataset of *C. siamense* s. lat. and related species (Additional file 11: Table S3). Only strains for which sequence information was available for all six loci were included in the dataset. Repeat haplotypes were removed from both single- and multi-locus phylogenetic analyses and the following species delimitation analyses. For comparison with previous studies, phylogenetic analysis (ML) was also calculated on the concatenated five-locus dataset (*CAL*, *GAPDH*, *GS*, *ITS* and *TUB2*) of the same strains.

#### Pairwise homoplasy index test

GCPSR is a pragmatic tool for the assessment of species limits, as the concordance of gene genealogies is a valuable criterion for evaluating the significance of gene flow between groups within an evolutionary timescale [71]. A pairwise homoplasy index (PHI) test using the GCPSR model was performed in SplitsTree4 [82, 83] to determine the recombination level between every pair of clades of *C. siamense* s. lat. Results of pairwise homoplasy index below a 0.05 threshold (Ф_w_ < 0.05) indicated significant recombination.

#### Phylogenetic network analysis

Phylogenetic network analysis is usually employed to infer evolutionary relationships when reticulate events such as hybridization, recombination and/or horizontal gene transfer are thought to be involved [84]. Single-locus ML trees of *C. siamense* s. lat. and related species were combined into single file and analyzed with Splitstree 4.10 [83] using SuperNetwork algorithms (Z-closure method, mintrees = 4, and 50 iterations).

### Coalescent-based species delimitation

To infer the species boundary of *C. siamense*, we first applied the General Mixed Yule Coalescent (GMYC) approach. This approach combines the neutral coalescent theory [85, 86] with the Yule speciation model [87] and aims at detecting shifts in branching rates between intra- and interspecific relationships. The ultrametric phylogenetic trees required to run the GMYC algorithm were created in BEAST v.1.8.1 [88] using unique haplotypes and the following parameters: GTR substitution model, site heterogeneity model of Gamma, random starting tree, and 5 × 10^7^ Markov Chain Monte Carlo (MCMC) generations sampled every 5,000 generations. Convergence was assessed by ESS values (≥200). A conservative burnin of 10 % was performed after checking the log-likelihood curves in Tracer v.1.6 [89]. We summarized the resulting trees into a target maximum clade credibility tree using TreeAnnotator v.1.8.1 [88]. The GMYC web server (The Exelixis Lab: http://species.hits.org/gmyc/) was used to fit our tree to both single-transition and multiple-transition GMYC models.

Secondly, the Poisson Tree Processes (PTP) model [90] was used to delimit species on a rooted phylogenetic tree. The PTP method estimates the mean expected number of substitutions per site between two branching events using the branch length information of a phylogeny and then implements two independent classes of poisson processes (intra and inter-specific branching events) before clustering the phylogenetic tree according to the results. The analysis was conducted on the web server for PTP (http://species.h-its.org/ptp/) using the RAxML tree as advocated for this method [90, 91].

Thirdly, a species validation method was applied. The posterior probability (PP) of inferred species was estimated using the program BPP (Bayesian Phylogenetics and Phylogeography) [36]. BPP is a Bayesian Markov Chain Monte Carlo (MCMC) program for analyzing DNA sequence alignments applying the multispecies coalescent model. This method accommodates the species phylogeny as well as incomplete lineage sorting due to ancestral polymorphism [36]. It has a number of advantages over other alternatives and is commonly used for species delimitation [92]. BPP v.3.1 incorporates nearest-neighbor interchange (NNI) algorithm allowing changes in the species tree topology and eliminating the need for a fixed user-specified guide tree [36]. Therefore we used the topology of the concatenated six-locus gene tree as guide tree for the BPP analyses. Four different sets of analyses with different values of α and β were conducted allowing *θ*s and τ_0_ to account for (i) large ancestral population sizes and deep divergence between species, G*θ*s (1, 10) and Gτ_0_ (1, 10), (ii) large ancestral population sizes and shallow divergences, G*θ*s (1, 10) and Gτ_0_ (2, 1000), (iii) small ancestral population sizes and shallow divergence, G*θ*s (2, 1000) and Gτ_0_ (2, 1000), and finally (iv) small ancestral population sizes and deep divergence, G*θ*s (2, 1000) and Gτ_0_ (1, 10). The analyses were performed with the following settings: species delimitation = 1, algorithm = 0, finetune ɛ = 2, usedata = 1 and cleandata = 0. The reversible-jump MCMC analyses consisted of 50,000 generations (sampling interval of 5) with 5,000 samples being discarded as burn-in. Each analysis was run twice using different starting seeds to confirm consistency between runs. With this approach, the validity of a speciation event is strongly supported if pp ≥ 0.95 [35].

### Morphological examination and mating test

Isolates of *C. siamense* s. lat. were cultivated on synthetic nutrient-poor agar medium (SNA) [93] amended with double-autoclaved pine needles placed onto the agar surface [94], and incubated at room temperature (c. 25 °C) in the dark. After two months, the cultures were examined under a Nikon SMZ1500 stereomicroscope for the presence of conidia and ascospores. The length and width of 40 conidia for each fertile strain were measured in lactic acid using a Nikon Eclipse 80i microscope. Average values were calculated and hierarchical clustering analysis (www.wessa.net) using the Ward’s method was carried out for the conidial length and width of *C. siamense* s. lat.

Eighteen of the strains that did not form a sexual morph were randomly selected to perform mating experiments. Mycelial plugs of each two parental strains were placed opposite each other and approximately 2 cm from the edge of 9 cm Petri dishes. Autoclaved pine needles were placed on the SNA between the two mycelia plugs to stimulate perithecial production. The plates were incubated at room temperature (ca. 25 °C) in the dark. After two months, the mating plates were examined for the presence of perithecia and ascospores.

### Ethics and consent to participate

Not applicable.

### Consent to publish

Not applicable.

### Availability of data and materials

The nucleic acid sequences supporting the results of this article are available in the GenBank repository, and all accession numbers are included in the Additional file 11. Supporting data sets are available in the electronic laboratory notebook LabArchives (https://mynotebook.labarchives.com/share/Data%2520of%2520EVOB-D-15-00473/MzIuNXwxNzEyNzAvMjUvVHJlZU5vZGUvNDE4MjUzOTYwOHw4Mi41, DOI: 10.6070/H40Z71BN, 10.6070/H4W66HTT, 10.6070/H4RF5S22, 10.6070/H4X63K02, 10.6070/H4MP5198, 10.6070/H4GX48M0, 10.6070/H4SF2T7W, 10.6070/H4C82799).

## Additional files

Additional file 1: Figure S1. Phylograms of *C. siamense* s. lat. resulted from the RAxML analyses based on the seven single loci, five-locus and eight-locus alignments, only bootstrap support value > 50 % are shown. Each isolate was marked with the’clade’ number that corresponds to the ApMat tree (Fig. 1). (PDF 1117 kb) Additional file 2: Table S1. Pairwise homoplasy index (PHI) of paired clades in *C. siamense* s. lat. (DOCX 17 kb) Additional file 3: Figure S2. Super-network obtained from the combined analyses of single-gene ML trees (ApMat, CAL, GAPDH, GS, ITS, TUB2). The scale indicates the mean distance obtained from the analysis of single-gene trees. (PDF 99 kb) Additional file 4: Figure S3. Ultrametric gene genealogy and clusters recognized by the single-threshold method of GMYC (Coalescent model). Putative species clusters are indicated using transitions between black-colored to red-colored branches. The inter- and intraspecific portions of the tree are divided with a vertical line. (PDF 214 kb) Additional file 5: Figure S4. Ultrametric gene genealogy and clusters recognized by the multi-threshold method of GMYC (Coalescent model). Putative species clusters are indicated using transitions between black-colored to red-colored branches. The inter- and intraspecific portions of the tree are divided with a vertical line. (PDF 207 kb) Additional file 6: Figure S5. Results of the PTP analysis based on the BI and ML topologies. Putative species clusters are indicated using transitions between blue-colored to red-colored branches. (PDF 422 kb) Additional file 7: Figure S6. Development of sexual structures through the interaction of isolates LC2937 × LC2875. a. mature perithecia. b, c. Asci and ascospores. Scale bars: b–c = 10 μm. (JPG 1761 kb) Additional file 8: Table S2. Sexual compatibility between isolates of *C. siamense* s. lat. (DOCX 18 kb) Additional file 9: Figure S7. Dendrogram resulted from the hierarchical clustering analysis with the Ward’s method showing the distribution of mean spore lengths and widths of isolatesof *C. siamense* s. lat. (PDF 109 kb) Additional file 10: Figure S8. Discordance between genes trees of ApMat (left) and 5-locus (CAL, GAPDH, GS, ITS, TUB2) (right) constructed with a maximum likelihood analysis by running RAxML v.7.0.3. The RAxML bootstrap support values (ML, >50) and Bayesian posterior probabilities (PP, >0.95) are displayed at the nodes (ML/PP). Ex-type cultures of described species within in *C. siamense* s. lat. indicated with red color. (PDF 485 kb) Additional file 11: Table S3. Details of isolates included in the phylogenetic analyses and species delimitation. (DOCX 39 kb) Additional file 12: Table S4 Primers used in this study, with sequences and sources. (DOCX 14 kb)
