# Supplementary material for: Species boundaries in plant pathogenic fungi: a Colletotrichum case study
Source: BMC Evol Biol. 2016 Apr 14;16:81. doi: 10.1186/s12862-016-0649-5 (PMC4832473; doi:10.1186/s12862-016-0649-5)
Supplement: Additional file 1: Figure S1. — Phylograms of C. siamense s. lat. resulted from the RAxML analyses based on the seven single loci, five-locus and eight-locus alignments, only bootstrap support value > 50 % are shown. Each isolate was marked with the’clade’ number that corresponds to the ApMat tree (Fig. 1). (PDF 1117 kb) [file 12862_2016_649_MOESM1_ESM.pdf]

Apn25L tree

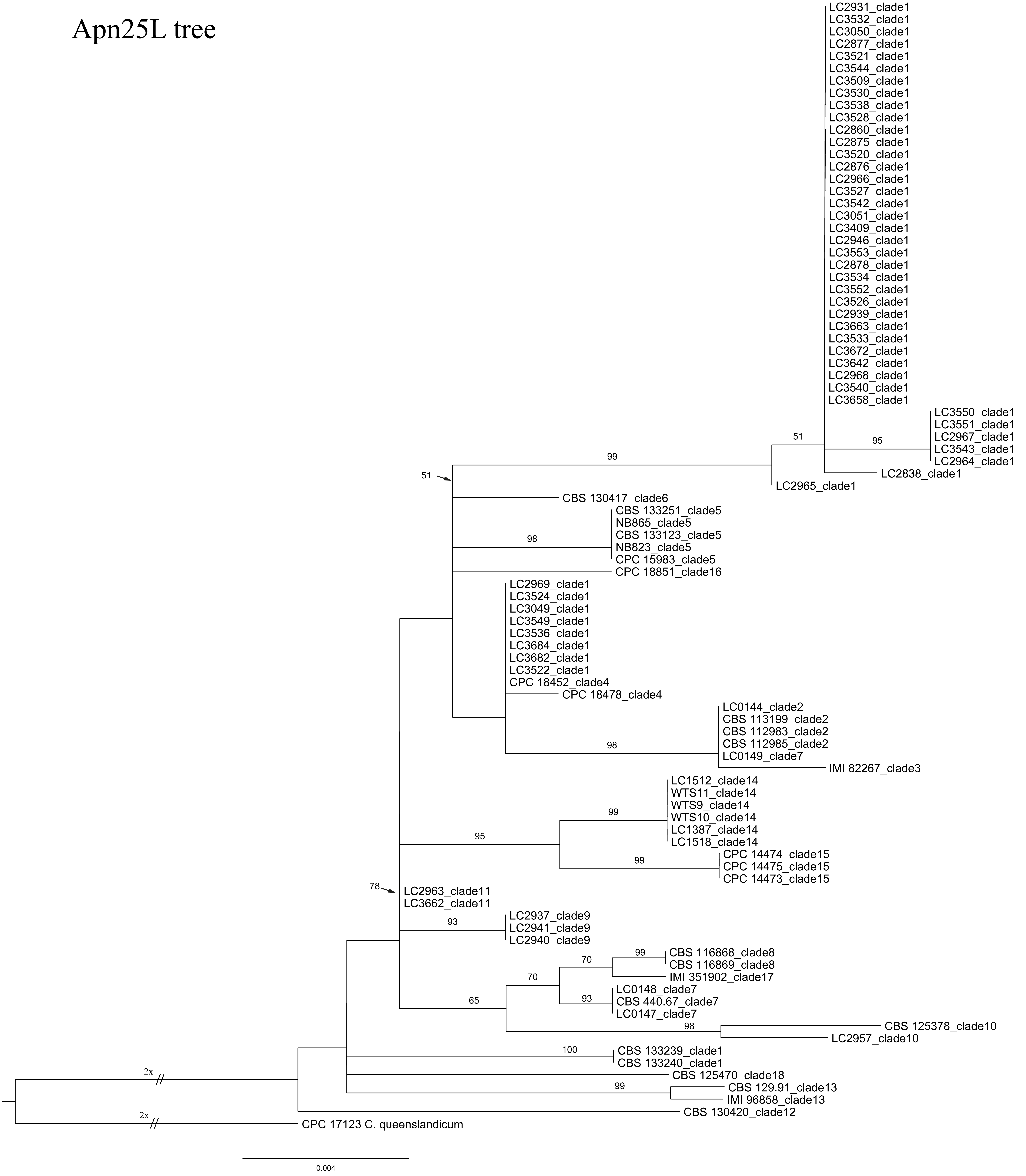

LC2931_clade1
LC3532_clade1
LC3050_clade1
LC2877_clade1
LC3521_clade1
LC3544_clade1
LC3509_clade1
LC3530_clade1
LC3538_clade1
LC3528_clade1
LC2860_clade1
LC2875_clade1
LC3520_clade1
LC2876_clade1
LC2966_clade1
LC3527_clade1
LC3542_clade1
LC3051_clade1
LC3409_clade1
LC2946_clade1
LC3553_clade1
LC2878_clade1
LC3534_clade1
LC3552_clade1
LC3526_clade1
LC2939_clade1
LC3663_clade1
LC3533_clade1
LC3672_clade1
LC3642_clade1
LC2968_clade1
LC3540_clade1
LC3658_clade1
LC3550_clade1
LC3551_clade1
LC2967_clade1
LC3543_clade1
LC2964_clade1
95
51
99
LC2838_clade1
51
LC2965_clade1
CBS 130417_clade6
CBS 133251_clade5
NB865_clade5
CBS 133123_clade5
NB823_clade5
CPC 15983_clade5
98
CPC 18851_clade16
LC2969_clade1
LC3524_clade1
LC3049_clade1
LC3549_clade1
LC3536_clade1
LC3684_clade1
LC3682_clade1
LC3522_clade1
CPC 18452_clade4
CPC 18478_clade4
LC0144_clade2
CBS 113199_clade2
CBS 112983_clade2
CBS 112985_clade2
LC0149_clade7
98
IMI 82267_clade3
LC1512_clade14
WTS11_clade14
WTS9_clade14
WTS10_clade14
LC1387_clade14
LC1518_clade14
99
95
CPC 14474_clade15
CPC 14475_clade15
CPC 14473_clade15
99
78
LC2963_clade11
LC3662_clade11
LC2937_clade9
LC2941_clade9
LC2940_clade9
93
99
CBS 116868_clade8
CBS 116869_clade8
70
IMI 351902_clade17
70
LC0148_clade7
CBS 440.67_clade7
93
LC0147_clade7
65
98
CBS 125378_clade10
LC2957_clade10
100
CBS 133239_clade1
CBS 133240_clade1
CBS 125470_clade18
99
CBS 129.91_clade13
IMI 96858_clade13
CBS 130420_clade12
2x
2x
CPC 17123 C. queenslandicum
0.004

CAL tree

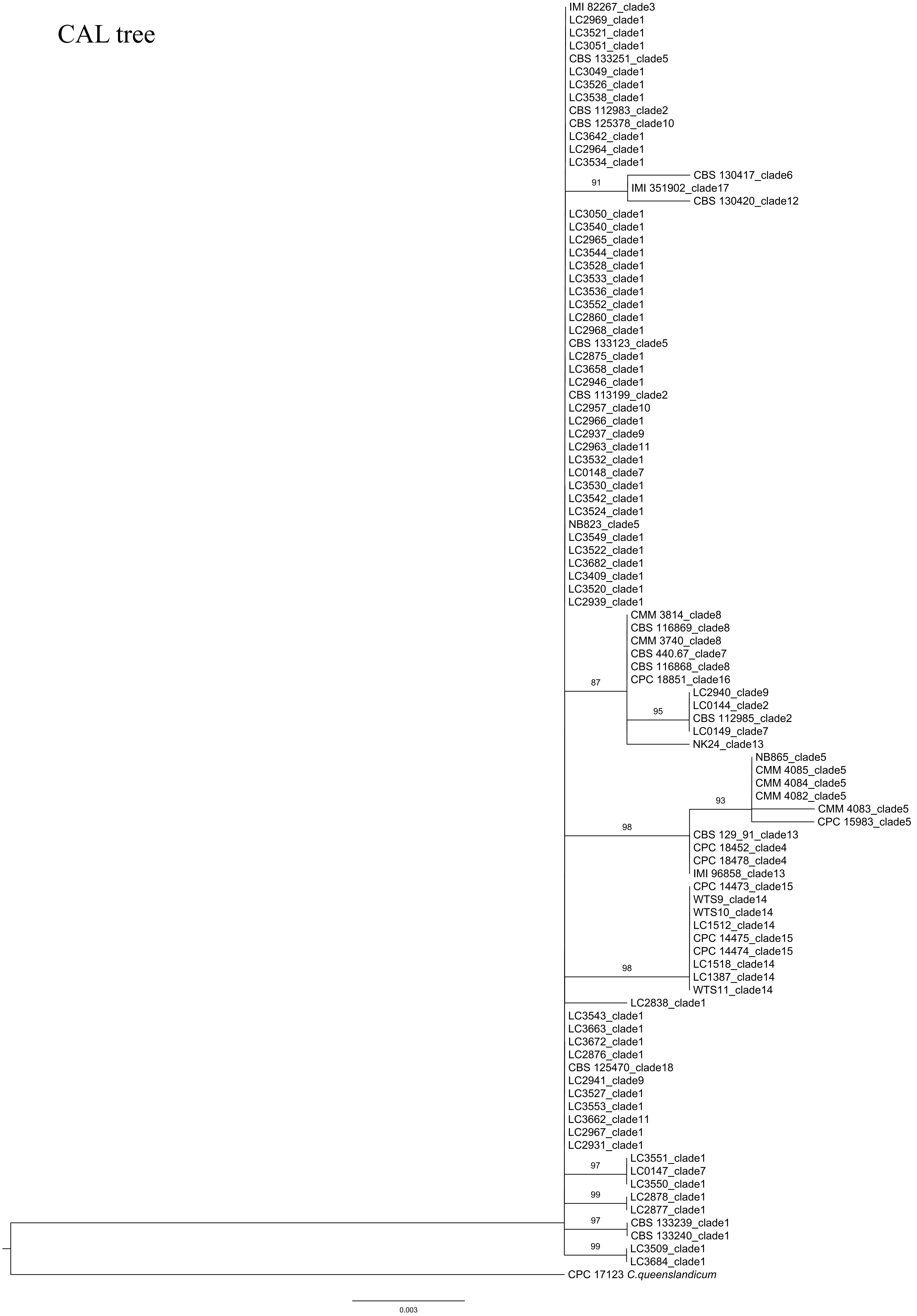
IMI 82267_clade3
LC2969_clade1
LC3521_clade1
LC3051_clade1
CBS 133251_clade5
LC3049_clade1
LC3526_clade1
LC3538_clade1
CBS 112983_clade2
CBS 125378_clade10
LC3642_clade1
LC2964_clade1
LC3534_clade1
91
CBS 130417_clade6
IMI 351902_clade17
CBS 130420_clade12
LC3050_clade1
LC3540_clade1
LC2965_clade1
LC3544_clade1
LC3528_clade1
LC3533_clade1
LC3536_clade1
LC3552_clade1
LC2860_clade1
LC2968_clade1
CBS 133123_clade5
LC2875_clade1
LC3658_clade1
LC2946_clade1
CBS 113199_clade2
LC2957_clade10
LC2966_clade1
LC2937_clade9
LC2963_clade11
LC3532_clade1
LC0148_clade7
LC3530_clade1
LC3542_clade1
LC3524_clade1
NB823_clade5
LC3549_clade1
LC3522_clade1
LC3682_clade1
LC3409_clade1
LC3520_clade1
LC2939_clade1
CMM 3814_clade8
CBS 116869_clade8
CMM 3740_clade8
CBS 440.67_clade7
CBS 116868_clade8
CPC 18851_clade16
87
LC2940_clade9
LC0144_clade2
95
CBS 112985_clade2
LC0149_clade7
NK24_clade13
NB865_clade5
CMM 4085_clade5
CMM 4084_clade5
CMM 4082_clade5
93
CMM 4083_clade5
CPC 15983_clade5
98
CBS 129_91_clade13
CPC 18452_clade4
CPC 18478_clade4
IMI 96858_clade13
CPC 14473_clade15
WTS9_clade14
WTS10_clade14
LC1512_clade14
CPC 14475_clade15
CPC 14474_clade15
98
LC1518_clade14
LC1387_clade14
WTS11_clade14
LC2838_clade1
LC3543_clade1
LC3663_clade1
LC3672_clade1
LC2876_clade1
CBS 125470_clade18
LC2941_clade9
LC3527_clade1
LC3553_clade1
LC3662_clade11
LC2967_clade1
LC2931_clade1
97
LC3551_clade1
LC0147_clade7
LC3550_clade1
99
LC2878_clade1
LC2877_clade1
97
CBS 133239_clade1
CBS 133240_clade1
99
LC3509_clade1
LC3684_clade1
CPC 17123 *C.queenslandicum*
0.003

GAPDH tree

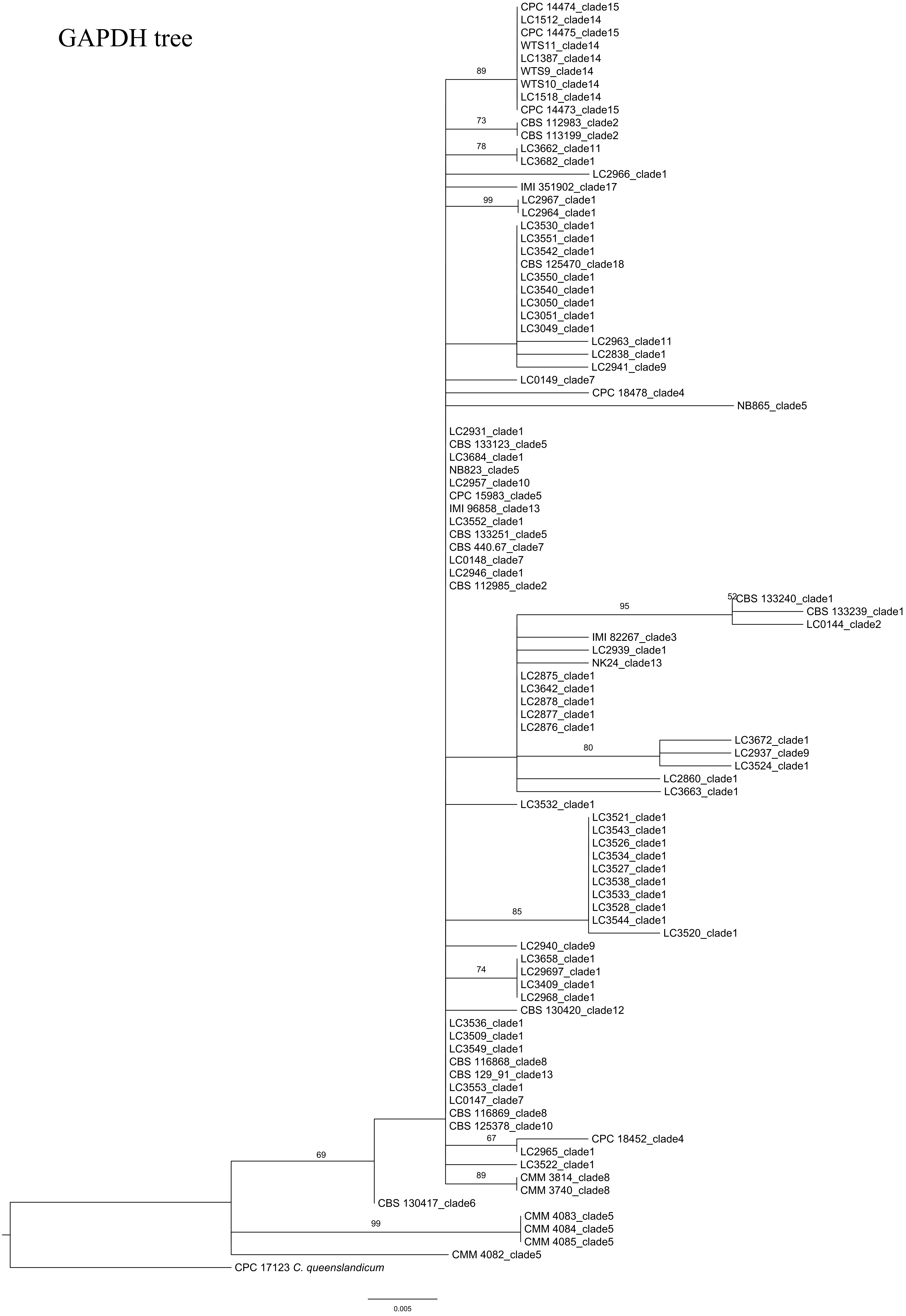

CPC 14474_clade15
LC1512_clade14
CPC 14475_clade15
WTS11_clade14
LC1387_clade14
WTS9_clade14
WTS10_clade14
LC1518_clade14
CPC 14473_clade15
89
73
CBS 112983_clade2
CBS 113199_clade2
78
LC3662_clade11
LC3682_clade1
LC2966_clade1
IMI 351902_clade17
99
LC2967_clade1
LC2964_clade1
LC3530_clade1
LC3551_clade1
LC3542_clade1
CBS 125470_clade18
LC3550_clade1
LC3540_clade1
LC3050_clade1
LC3051_clade1
LC3049_clade1
LC2963_clade11
LC2838_clade1
LC2941_clade9
LC0149_clade7
CPC 18478_clade4
NB865_clade5
LC2931_clade1
CBS 133123_clade5
LC3684_clade1
NB823_clade5
LC2957_clade10
CPC 15983_clade5
IMI 96858_clade13
LC3552_clade1
CBS 133251_clade5
CBS 440.67_clade7
LC0148_clade7
LC2946_clade1
CBS 112985_clade2
95
52
CBS 133240_clade1
CBS 133239_clade1
LC0144_clade2
IMI 82267_clade3
LC2939_clade1
NK24_clade13
LC2875_clade1
LC3642_clade1
LC2878_clade1
LC2877_clade1
LC2876_clade1
80
LC3672_clade1
LC2937_clade9
LC3524_clade1
LC2860_clade1
LC3663_clade1
LC3532_clade1
LC3521_clade1
LC3543_clade1
LC3526_clade1
LC3534_clade1
LC3527_clade1
LC3538_clade1
LC3533_clade1
LC3528_clade1
LC3544_clade1
85
LC3520_clade1
LC2940_clade9
74
LC3658_clade1
LC29697_clade1
LC3409_clade1
LC2968_clade1
CBS 130420_clade12
LC3536_clade1
LC3509_clade1
LC3549_clade1
CBS 116868_clade8
CBS 129_91_clade13
LC3553_clade1
LC0147_clade7
CBS 116869_clade8
CBS 125378_clade10
67
CPC 18452_clade4
LC2965_clade1
LC3522_clade1
89
CMM 3814_clade8
CMM 3740_clade8
69
CBS 130417_clade6
99
CMM 4083_clade5
CMM 4084_clade5
CMM 4085_clade5
CMM 4082_clade5
CPC 17123 *C. queenslandicum*
0.005

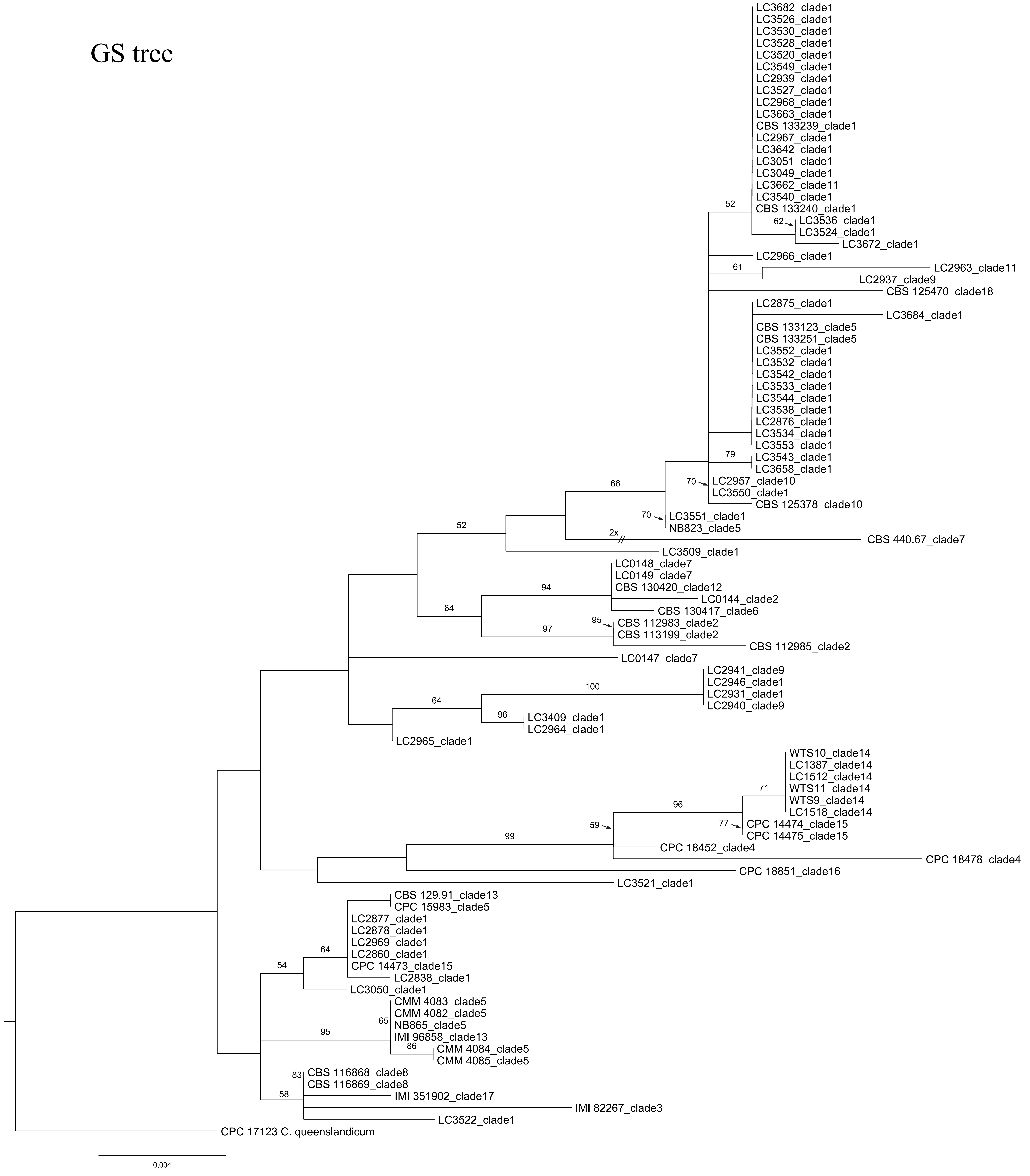
GS tree
LC3682_clade1
LC3526_clade1
LC3530_clade1
LC3528_clade1
LC3520_clade1
LC3549_clade1
LC2939_clade1
LC3527_clade1
LC2968_clade1
LC3663_clade1
CBS 133239_clade1
LC2967_clade1
LC3642_clade1
LC3051_clade1
LC3049_clade1
LC3662_clade11
LC3540_clade1
52
CBS 133240_clade1
62
LC3536_clade1
LC3524_clade1
LC3672_clade1
LC2966_clade1
61
LC2963_clade11
LC2937_clade9
CBS 125470_clade18
LC2875_clade1
LC3684_clade1
CBS 133123_clade5
CBS 133251_clade5
LC3552_clade1
LC3532_clade1
LC3542_clade1
LC3533_clade1
LC3544_clade1
LC3538_clade1
LC2876_clade1
LC3534_clade1
LC3553_clade1
79
LC3543_clade1
LC3658_clade1
70
LC2957_clade10
LC3550_clade1
66
CBS 125378_clade10
70
LC3551_clade1
NB823_clade5
52
2x
CBS 440.67_clade7
LC3509_clade1
LC0148_clade7
LC0149_clade7
94
CBS 130420_clade12
LC0144_clade2
64
CBS 130417_clade6
95
CBS 112983_clade2
97
CBS 113199_clade2
CBS 112985_clade2
LC0147_clade7
LC2941_clade9
100
LC2946_clade1
LC2931_clade1
64
LC2940_clade9
96
LC3409_clade1
LC2964_clade1
LC2965_clade1
WTS10_clade14
LC1387_clade14
LC1512_clade14
71
WTS11_clade14
96
WTS9_clade14
LC1518_clade14
77
CPC 14474_clade15
59
99
CPC 14475_clade15
CPC 18452_clade4
CPC 18478_clade4
CPC 18851_clade16
LC3521_clade1
CBS 129.91_clade13
CPC 15983_clade5
LC2877_clade1
LC2878_clade1
LC2969_clade1
64
LC2860_clade1
54
CPC 14473_clade15
LC2838_clade1
LC3050_clade1
CMM 4083_clade5
CMM 4082_clade5
65
NB865_clade5
95
IMI 96858_clade13
86
CMM 4084_clade5
CMM 4085_clade5
83
CBS 116868_clade8
CBS 116869_clade8
58
IMI 351902_clade17
IMI 82267_clade3
LC3522_clade1
CPC 17123 C. queenslandicum
0.004

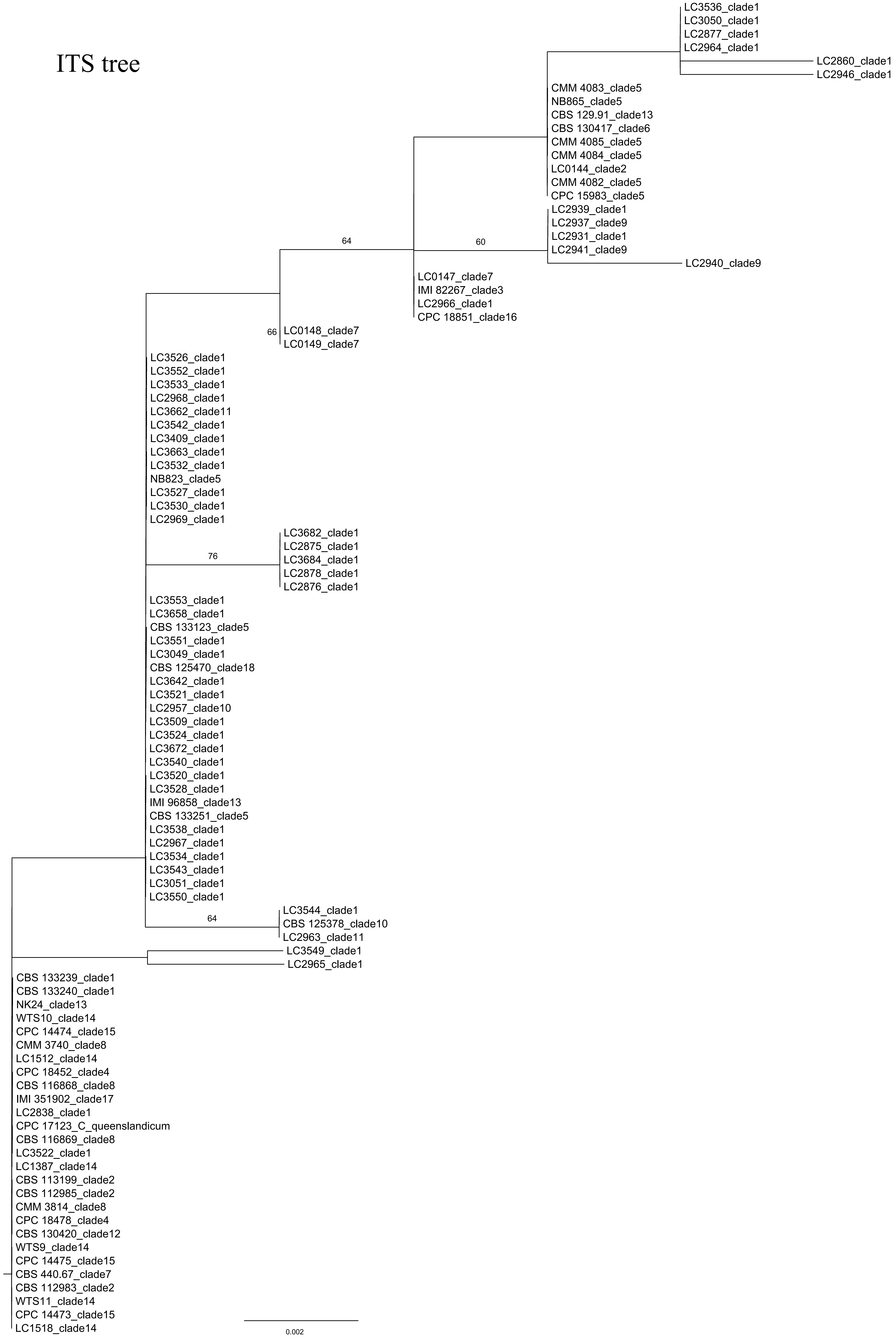
ITS tree
LC3536_clade1
LC3050_clade1
LC2877_clade1
LC2964_clade1
LC2860_clade1
LC2946_clade1
CMM 4083_clade5
NB865_clade5
CBS 129.91_clade13
CBS 130417_clade6
CMM 4085_clade5
CMM 4084_clade5
LC0144_clade2
CMM 4082_clade5
CPC 15983_clade5
LC2939_clade1
LC2937_clade9
LC2931_clade1
LC2941_clade9
64
60
LC2940_clade9
LC0147_clade7
IMI 82267_clade3
LC2966_clade1
CPC 18851_clade16
66
LC0148_clade7
LC0149_clade7
LC3526_clade1
LC3552_clade1
LC3533_clade1
LC2968_clade1
LC3662_clade11
LC3542_clade1
LC3409_clade1
LC3663_clade1
LC3532_clade1
NB823_clade5
LC3527_clade1
LC3530_clade1
LC2969_clade1
LC3682_clade1
LC2875_clade1
76
LC3684_clade1
LC2878_clade1
LC2876_clade1
LC3553_clade1
LC3658_clade1
CBS 133123_clade5
LC3551_clade1
LC3049_clade1
CBS 125470_clade18
LC3642_clade1
LC3521_clade1
LC2957_clade10
LC3509_clade1
LC3524_clade1
LC3672_clade1
LC3540_clade1
LC3520_clade1
LC3528_clade1
IMI 96858_clade13
CBS 133251_clade5
LC3538_clade1
LC2967_clade1
LC3534_clade1
LC3543_clade1
LC3051_clade1
LC3550_clade1
LC3544_clade1
64
CBS 125378_clade10
LC2963_clade11
LC3549_clade1
LC2965_clade1
CBS 133239_clade1
CBS 133240_clade1
NK24_clade13
WTS10_clade14
CPC 14474_clade15
CMM 3740_clade8
LC1512_clade14
CPC 18452_clade4
CBS 116868_clade8
IMI 351902_clade17
LC2838_clade1
CPC 17123_C_queenslandicum
CBS 116869_clade8
LC3522_clade1
LC1387_clade14
CBS 113199_clade2
CBS 112985_clade2
CMM 3814_clade8
CPC 18478_clade4
CBS 130420_clade12
WTS9_clade14
CPC 14475_clade15
CBS 440.67_clade7
CBS 112983_clade2
WTS11_clade14
CPC 14473_clade15
LC1518_clade14
0.002

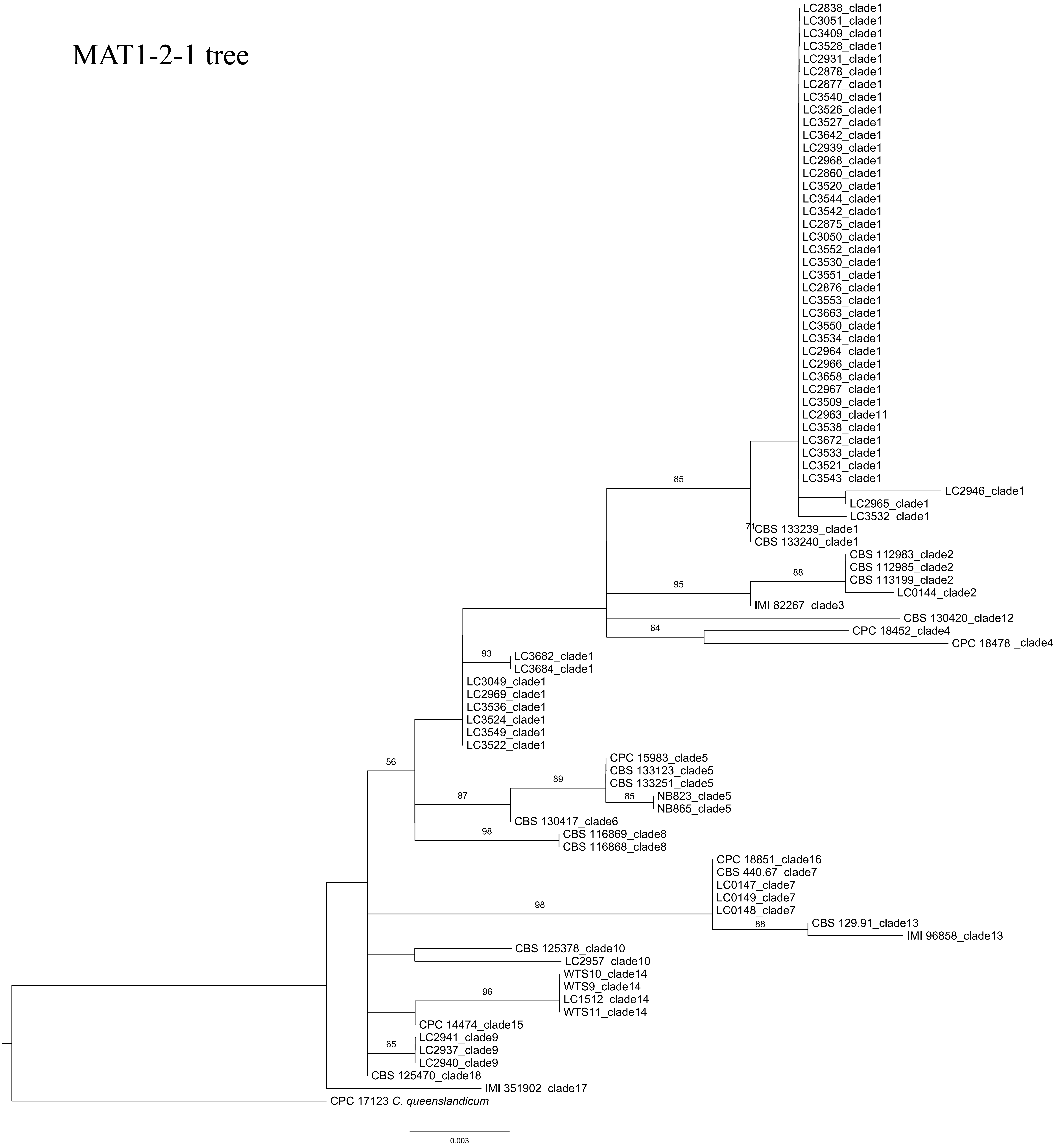
MAT1-2-1 tree
LC2838_clade1
LC3051_clade1
LC3409_clade1
LC3528_clade1
LC2931_clade1
LC2878_clade1
LC2877_clade1
LC3540_clade1
LC3526_clade1
LC3527_clade1
LC3642_clade1
LC2939_clade1
LC2968_clade1
LC2860_clade1
LC3520_clade1
LC3544_clade1
LC3542_clade1
LC2875_clade1
LC3050_clade1
LC3552_clade1
LC3530_clade1
LC3551_clade1
LC2876_clade1
LC3553_clade1
LC3663_clade1
LC3550_clade1
LC3534_clade1
LC2964_clade1
LC2966_clade1
LC3658_clade1
LC2967_clade1
LC3509_clade1
LC2963_clade11
LC3538_clade1
LC3672_clade1
LC3533_clade1
LC3521_clade1
LC3543_clade1
LC2946_clade1
LC2965_clade1
LC3532_clade1
85
71
CBS 133239_clade1
CBS 133240_clade1
CBS 112983_clade2
CBS 112985_clade2
CBS 113199_clade2
88
95
LC0144_clade2
IMI 82267_clade3
CBS 130420_clade12
64
CPC 18452_clade4
CPC 18478 _clade4
93
LC3682_clade1
LC3684_clade1
LC3049_clade1
LC2969_clade1
LC3536_clade1
LC3524_clade1
LC3549_clade1
LC3522_clade1
56
CPC 15983_clade5
CBS 133123_clade5
CBS 133251_clade5
89
87
85
NB823_clade5
NB865_clade5
CBS 130417_clade6
98
CBS 116869_clade8
CBS 116868_clade8
CPC 18851_clade16
CBS 440.67_clade7
LC0147_clade7
LC0149_clade7
LC0148_clade7
98
88
CBS 129.91_clade13
IMI 96858_clade13
CBS 125378_clade10
LC2957_clade10
WTS10_clade14
WTS9_clade14
96
LC1512_clade14
WTS11_clade14
CPC 14474_clade15
65
LC2941_clade9
LC2937_clade9
LC2940_clade9
CBS 125470_clade18
IMI 351902_clade17
CPC 17123 C. queenslandicum
0.003

TUB2 tree

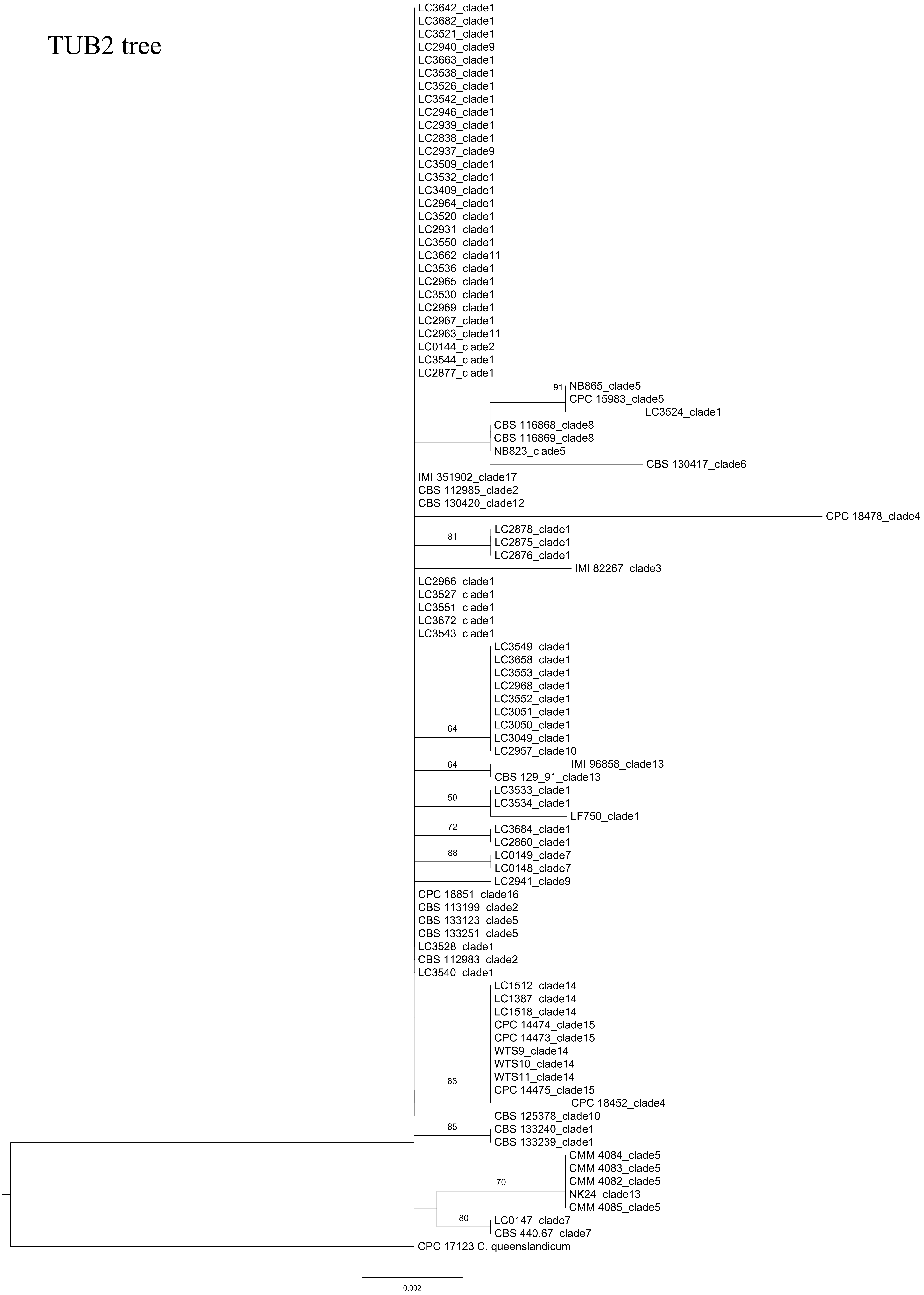
LC3642_clade1
LC3682_clade1
LC3521_clade1
LC2940_clade9
LC3663_clade1
LC3538_clade1
LC3526_clade1
LC3542_clade1
LC2946_clade1
LC2939_clade1
LC2838_clade1
LC2937_clade9
LC3509_clade1
LC3532_clade1
LC3409_clade1
LC2964_clade1
LC3520_clade1
LC2931_clade1
LC3550_clade1
LC3662_clade11
LC3536_clade1
LC2965_clade1
LC3530_clade1
LC2969_clade1
LC2967_clade1
LC2963_clade11
LC0144_clade2
LC3544_clade1
LC2877_clade1
91
NB865_clade5
CPC 15983_clade5
LC3524_clade1
CBS 116868_clade8
CBS 116869_clade8
NB823_clade5
CBS 130417_clade6
IMI 351902_clade17
CBS 112985_clade2
CBS 130420_clade12
CPC 18478_clade4
81
LC2878_clade1
LC2875_clade1
LC2876_clade1
IMI 82267_clade3
LC2966_clade1
LC3527_clade1
LC3551_clade1
LC3672_clade1
LC3543_clade1
LC3549_clade1
LC3658_clade1
LC3553_clade1
LC2968_clade1
LC3552_clade1
LC3051_clade1
64
LC3050_clade1
LC3049_clade1
LC2957_clade10
64
IMI 96858_clade13
CBS 129_91_clade13
50
LC3533_clade1
LC3534_clade1
LF750_clade1
72
LC3684_clade1
LC2860_clade1
88
LC0149_clade7
LC0148_clade7
LC2941_clade9
CPC 18851_clade16
CBS 113199_clade2
CBS 133123_clade5
CBS 133251_clade5
LC3528_clade1
CBS 112983_clade2
LC3540_clade1
LC1512_clade14
LC1387_clade14
LC1518_clade14
CPC 14474_clade15
CPC 14473_clade15
WTS9_clade14
WTS10_clade14
WTS11_clade14
63
CPC 14475_clade15
CPC 18452_clade4
CBS 125378_clade10
85
CBS 133240_clade1
CBS 133239_clade1
CMM 4084_clade5
CMM 4083_clade5
70
CMM 4082_clade5
NK24_clade13
CMM 4085_clade5
80
LC0147_clade7
CBS 440.67_clade7
CPC 17123 C. queenslandicum
0.002

CAL-GAPDH-GS-ITS-TUB2 tree

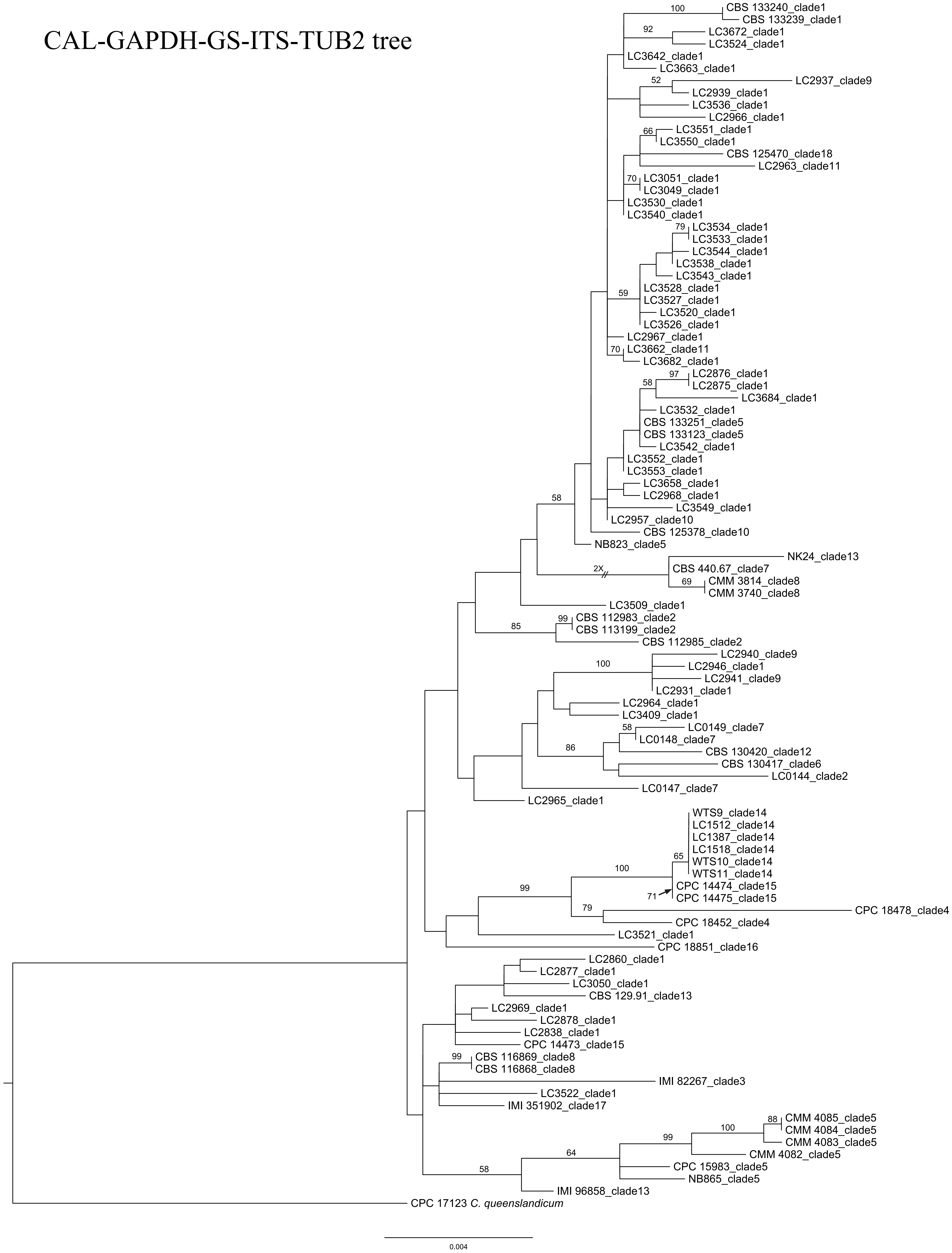

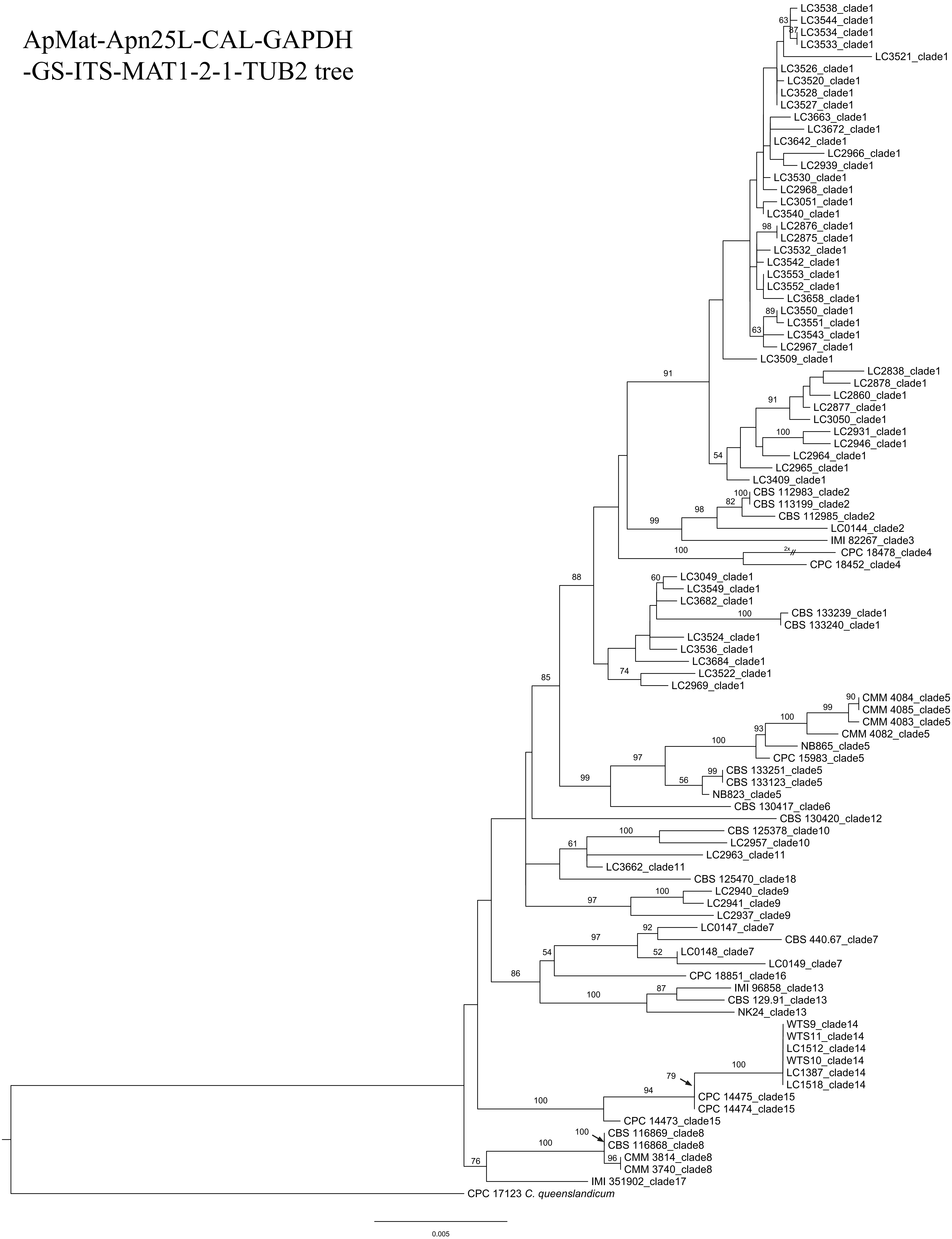
ApMat-Apn25L-CAL-GAPDH
-GS-ITS-MAT1-2-1-TUB2 tree
LC3538_clade1
LC3544_clade1
LC3534_clade1
LC3533_clade1
LC3521_clade1
LC3526_clade1
LC3520_clade1
LC3528_clade1
LC3527_clade1
LC3663_clade1
LC3672_clade1
LC3642_clade1
LC2966_clade1
LC2939_clade1
LC3530_clade1
LC2968_clade1
LC3051_clade1
LC3540_clade1
LC2876_clade1
LC2875_clade1
LC3532_clade1
LC3542_clade1
LC3553_clade1
LC3552_clade1
LC3658_clade1
LC3550_clade1
LC3551_clade1
LC3543_clade1
LC2967_clade1
LC3509_clade1
LC2838_clade1
LC2878_clade1
LC2860_clade1
LC2877_clade1
LC3050_clade1
LC2931_clade1
LC2946_clade1
LC2964_clade1
LC2965_clade1
LC3409_clade1
CBS 112983_clade2
CBS 113199_clade2
CBS 112985_clade2
LC0144_clade2
IMI 82267_clade3
CPC 18478_clade4
CPC 18452_clade4
LC3049_clade1
LC3549_clade1
LC3682_clade1
CBS 133239_clade1
CBS 133240_clade1
LC3524_clade1
LC3536_clade1
LC3684_clade1
LC3522_clade1
LC2969_clade1
CMM 4084_clade5
CMM 4085_clade5
CMM 4083_clade5
CMM 4082_clade5
NB865_clade5
CPC 15983_clade5
CBS 133251_clade5
CBS 133123_clade5
NB823_clade5
CBS 130417_clade6
CBS 130420_clade12
CBS 125378_clade10
LC2957_clade10
LC2963_clade11
LC3662_clade11
CBS 125470_clade18
LC2940_clade9
LC2941_clade9
LC2937_clade9
LC0147_clade7
CBS 440.67_clade7
LC0148_clade7
LC0149_clade7
CPC 18851_clade16
IMI 96858_clade13
CBS 129.91_clade13
NK24_clade13
WTS9_clade14
WTS11_clade14
LC1512_clade14
WTS10_clade14
LC1387_clade14
LC1518_clade14
CPC 14475_clade15
CPC 14474_clade15
CPC 14473_clade15
CBS 116869_clade8
CBS 116868_clade8
CMM 3814_clade8
CMM 3740_clade8
IMI 351902_clade17
CPC 17123 *C. queenslandicum*
0.005
