# Supplementary figures and images for: Species boundaries in plant pathogenic fungi: a Colletotrichum case study
Source: BMC Evol Biol. 2016 Apr 14;16:81. doi: 10.1186/s12862-016-0649-5 (PMC4832473; doi:10.1186/s12862-016-0649-5)

1.0E-5

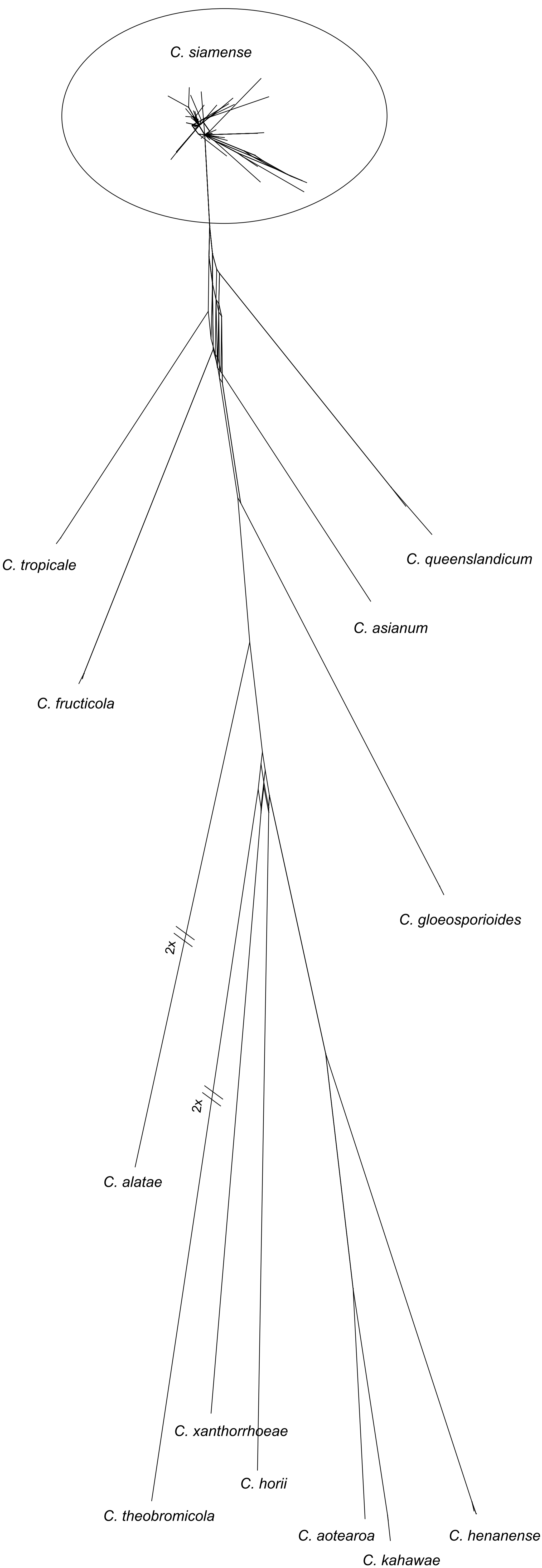

Supplement: Additional file 3: Figure S2. — Super-network obtained from the combined analyses of single-gene ML trees (ApMat, CAL, GAPDH, GS, ITS, TUB2). The scale indicates the mean distance obtained from the analysis of single-gene trees. (PDF 99 kb) [file 12862_2016_649_MOESM3_ESM.pdf]

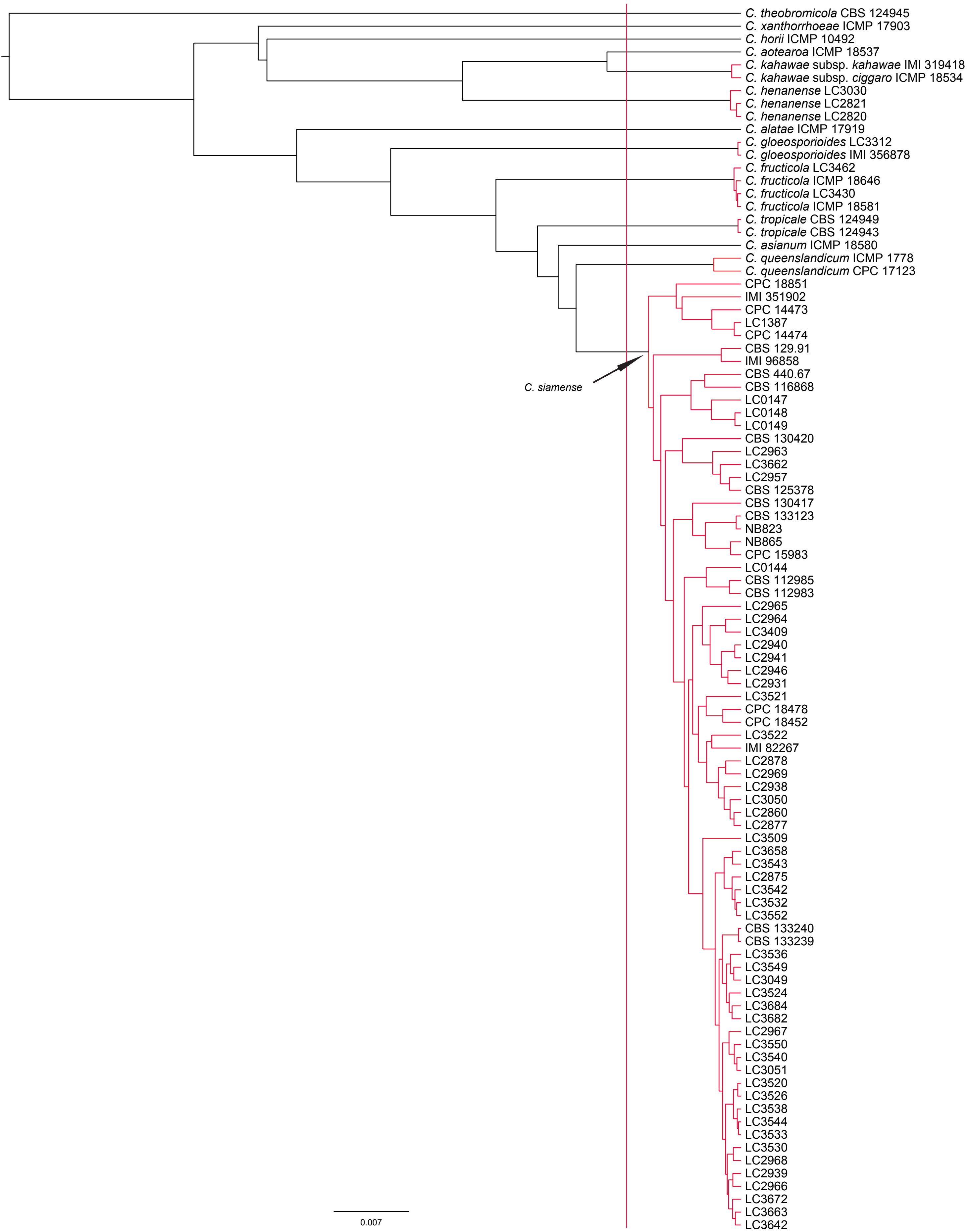

Supplement: Additional file 4: Figure S3. — Ultrametric gene genealogy and clusters recognized by the single-threshold method of GMYC (Coalescent model). Putative species clusters are indicated using transitions between black-colored to red-colored branches. The inter- and intraspecific portions of the tree are divided with a vertical line. (PDF 214 kb) [file 12862_2016_649_MOESM4_ESM.pdf]

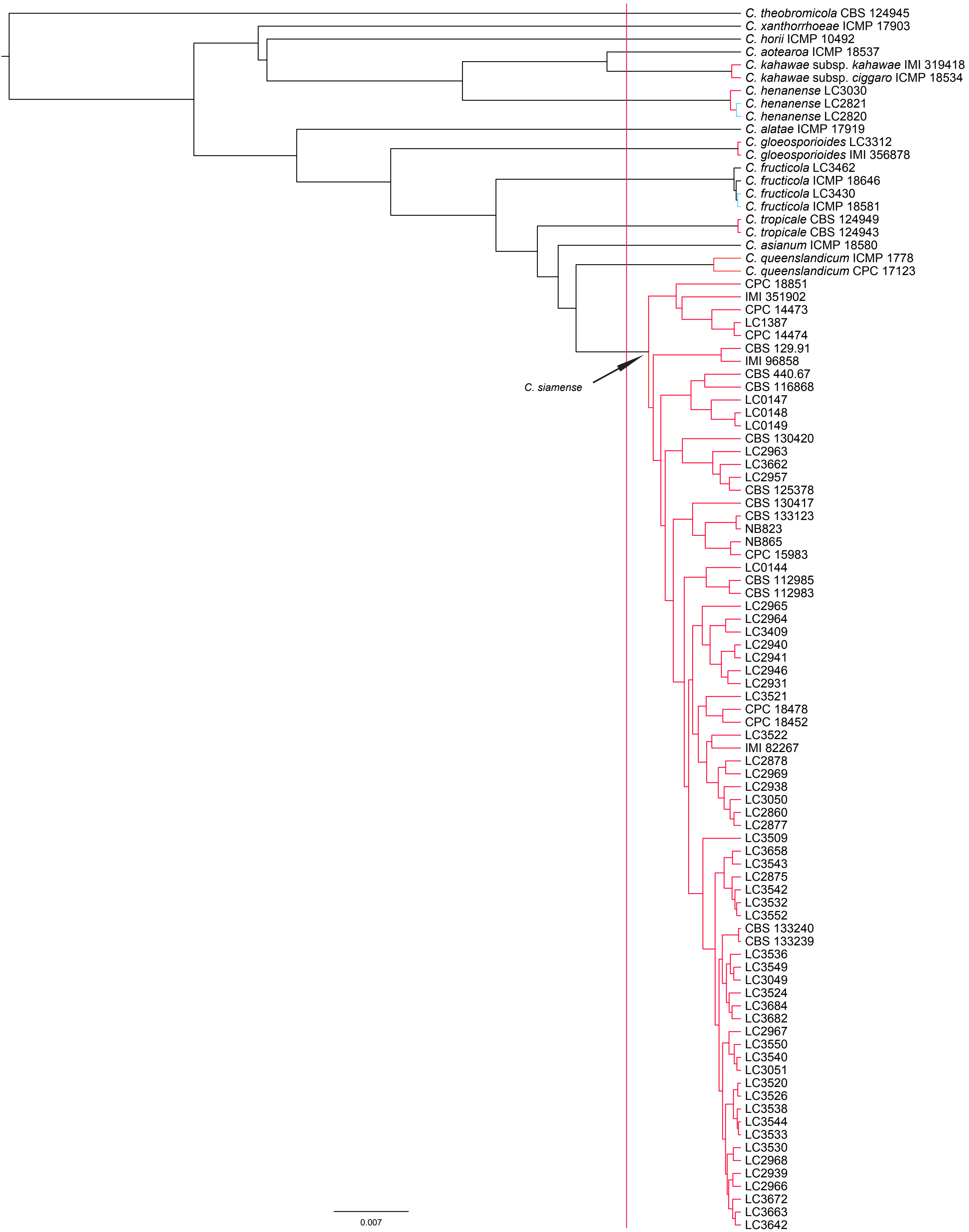

Supplement: Additional file 5: Figure S4. — Ultrametric gene genealogy and clusters recognized by the multi-threshold method of GMYC (Coalescent model). Putative species clusters are indicated using transitions between black-colored to red-colored branches. The inter- and intraspecific portions of the tree are divided with a vertical line. (PDF 207 kb) [file 12862_2016_649_MOESM5_ESM.pdf]

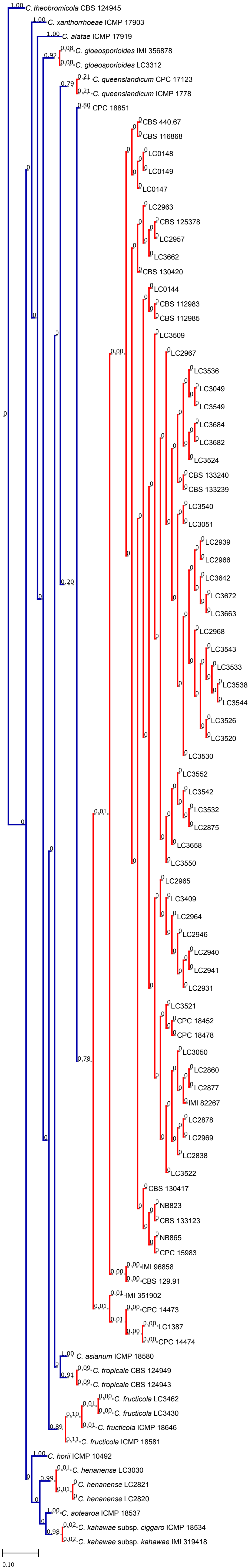

0.10

Supplement: Additional file 6: Figure S5. — Results of the PTP analysis based on the BI and ML topologies. Putative species clusters are indicated using transitions between blue-colored to red-colored branches. (PDF 422 kb) [file 12862_2016_649_MOESM6_ESM.pdf]

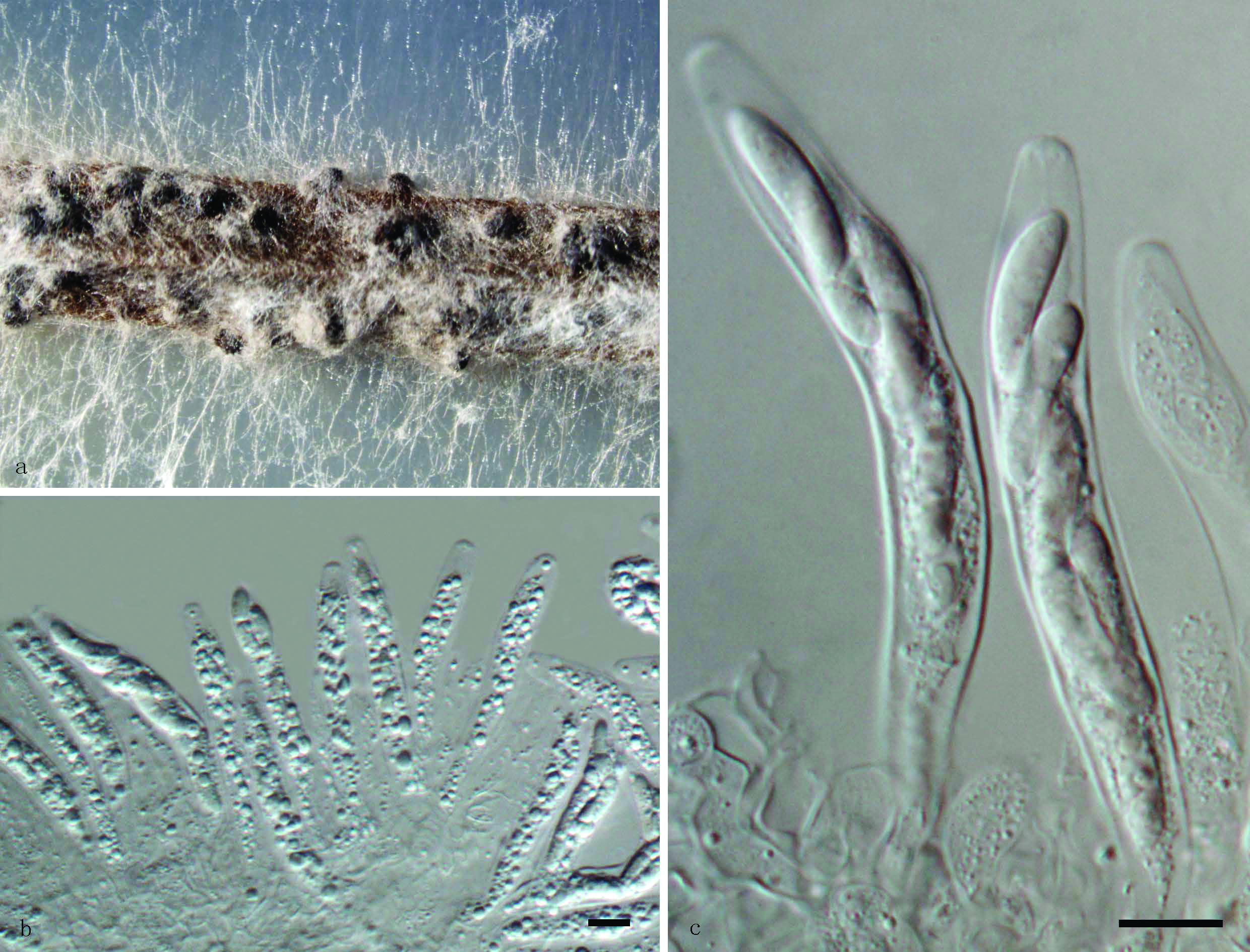

Supplement: Additional file 7: Figure S6. — Development of sexual structures through the interaction of isolates LC2937 × LC2875. a. mature perithecia. b, c. Asci and ascospores. Scale bars: b–c = 10 μm. (JPG 1761 kb) [file 12862_2016_649_MOESM7_ESM.jpg]

CPC 0117123 *C. queenslandicum*

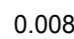

0.004

Supplement: Additional file 10: Figure S8. — Discordance between genes trees of ApMat (left) and 5-locus (CAL, GAPDH, GS, ITS, TUB2) (right) constructed with a maximum likelihood analysis by running RAxML v.7.0.3. The RAxML bootstrap support values (ML, >50) and Bayesian posterior probabilities (PP, >0.95) are displayed at the nodes (ML/PP). Ex-type cultures of described species within in C. siamense s. lat. indicated with red color. (PDF 485 kb) [file 12862_2016_649_MOESM10_ESM.pdf]
