# Supplementary material for: Species boundaries in plant pathogenic fungi: a Colletotrichum case study
Source: BMC Evol Biol. 2016 Apr 14;16:81. doi: 10.1186/s12862-016-0649-5 (PMC4832473; doi:10.1186/s12862-016-0649-5)
Supplement: Additional file 8: Table S2. — Sexual compatibility between isolates of C. siamense s. lat. (DOCX 18 kb) [file 12862_2016_649_MOESM8_ESM.docx]

Table S4 Sexual compatibility between isolates of *C. siamense* s. lat.

| Isolate | LC2838  clade 1 | LC2875 clade 1 | LC2931 clade 1 | LC2969 clade 1 | LC3050 clade 1 | LC3538 clade 1 | LC3543 clade 1 | LC3642 clade 1 | LC3682  clade 1 | CPC15983 clade 5 | CBS133123 clade 5 | LC0147 clade 7 | LC0148 clade 7 | LC2937 clade 9 | LC2957 clade 10 | LC3662 clade 11 | LC1387 clade 14 | LC1518 clade 14 |
| --- | --- | --- | --- | --- | --- | --- | --- | --- | --- | --- | --- | --- | --- | --- | --- | --- | --- | --- |
| LC2838_clade 1 | - |  |  |  |  |  |  |  |  |  |  |  |  |  |  |  |  |  |
| LC2875_clade 1 | + | - |  |  |  |  |  |  |  |  |  |  |  |  |  |  |  |  |
| LC2931_clade 1 | + | * | - |  |  |  |  |  |  |  |  |  |  |  |  |  |  |  |
| LC2969_clade 1 | - | * | - | - |  |  |  |  |  |  |  |  |  |  |  |  |  |  |
| LC3050_clade 1 | - | + | + | - | - |  |  |  |  |  |  |  |  |  |  |  |  |  |
| LC3538_clade 1 | - | * | + | - | + | - |  |  |  |  |  |  |  |  |  |  |  |  |
| LC3543_clade 1 | + | + | * | * | + | * | - |  |  |  |  |  |  |  |  |  |  |  |
| LC3642_clade 1 | + | + | + | - | - | + | * | - |  |  |  |  |  |  |  |  |  |  |
| LC3682_clade 1 | + | * | + | + | - | - | * | - | - |  |  |  |  |  |  |  |  |  |
| CPC15983_clade 5 | - | * | * | * | - | * | * | + | * | - |  |  |  |  |  |  |  |  |
| CBS133123_clade 5 | * | * | * | * | * | * | * | + | * | * | - |  |  |  |  |  |  |  |
| LC0147_clade 7 | - | + | + | - | - | + | - | - | - | * | * | - |  |  |  |  |  |  |
| LC0148_clade 7 | + | - | + | - | + | - | - | + | - | * | * | + | - |  |  |  |  |  |
| LC2937_clade 9 | + | - | + | + | + | + | + | - | + | * | * | * | + | - |  |  |  |  |
| LC2957_clade 10 | + | * | * | * | * | * | * | * | * | * | * | * | * | * | - |  |  |  |
| LC3662_clade 11 | + | + | + | - | + | - | - | + | + | * | * | * | * | * | * | - |  |  |
| LC1387_clade 14 | - | - | - | - | - | + | - | * | - | * | * | * | * | * | * | * | - |  |
| LC1518_clade 14 | - | - | - | - | - | - | - | - | - | * | * | * | * | * | * | * | + | - |

(-) ﹦ Negative: Mycelial growth with or without production of acervuli; (+) ﹦ Positive: Perithecia and ascospores present with acervuli; (*) ﹦ Not tested
