# Supplementary material for: Species boundaries in plant pathogenic fungi: a Colletotrichum case study
Source: BMC Evol Biol. 2016 Apr 14;16:81. doi: 10.1186/s12862-016-0649-5 (PMC4832473; doi:10.1186/s12862-016-0649-5)
Supplement: Additional file 9: Figure S7. — Dendrogram resulted from the hierarchical clustering analysis with the Ward’s method showing the distribution of mean spore lengths and widths of isolatesof C. siamense s. lat. (PDF 109 kb) [file 12862_2016_649_MOESM9_ESM.pdf]

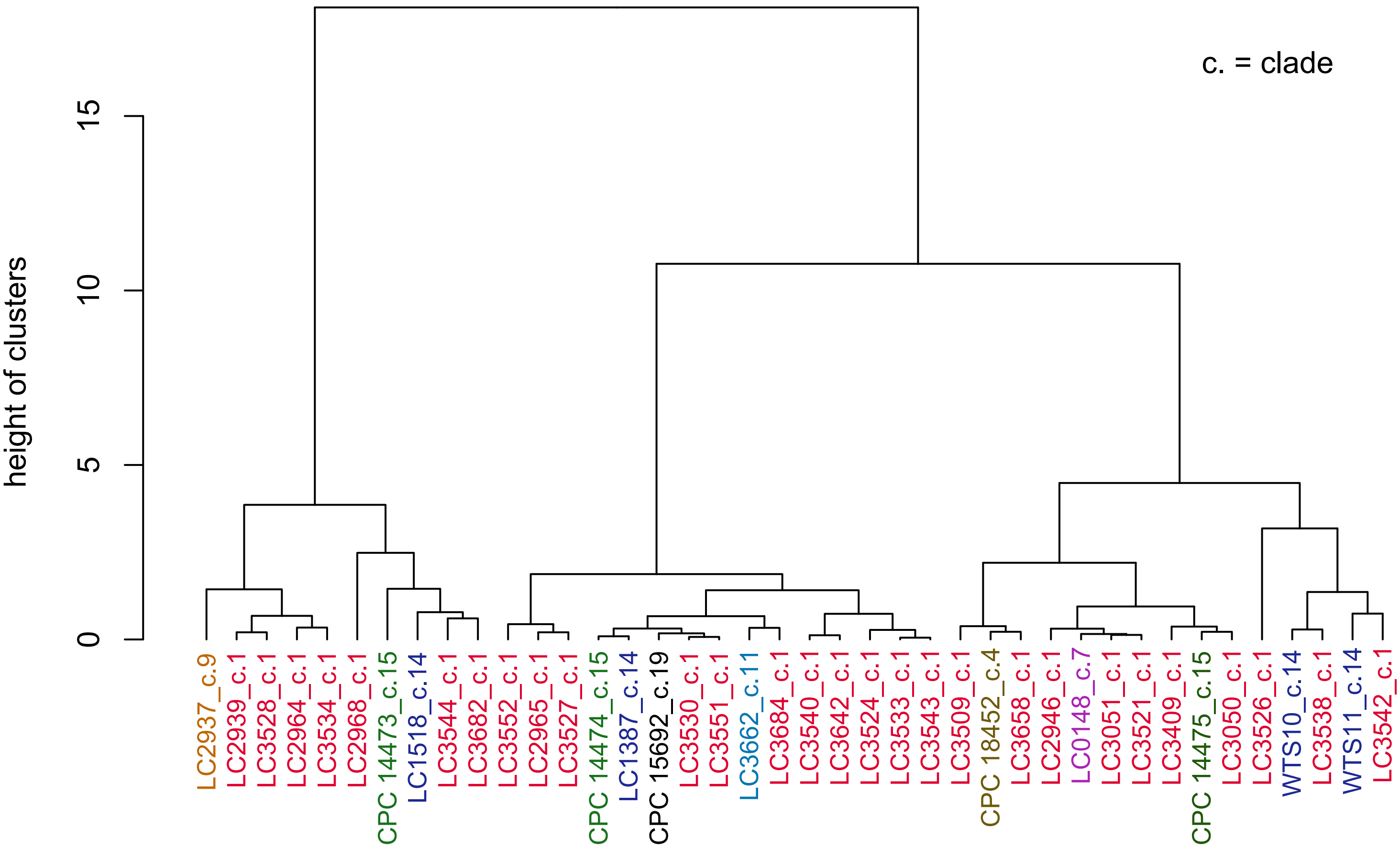
c. = clade
height of clusters
0
5
10
15
LC2937_c.9
LC2939_c.1
LC3528_c.1
LC2964_c.1
LC3534_c.1
LC2968_c.1
CPC 14473_c.15
LC1518_c.14
LC3544_c.1
LC3682_c.1
LC3552_c.1
LC2965_c.1
LC3527_c.1
CPC 14474_c.15
LC1387_c.14
CPC 15692_c.19
LC3530_c.1
LC3551_c.1
LC3662_c.11
LC3684_c.1
LC3540_c.1
LC3642_c.1
LC3524_c.1
LC3533_c.1
LC3543_c.1
LC3509_c.1
CPC 18452_c.4
LC3658_c.1
LC2946_c.1
LC0148_c.7
LC3051_c.1
LC3521_c.1
LC3409_c.1
CPC 14475_c.15
LC3050_c.1
LC3526_c.1
WTS10_c.14
LC3538_c.1
WTS11_c.14
LC3542_c.1
