# Supplementary material for: Species boundaries in plant pathogenic fungi: a Colletotrichum case study
Source: BMC Evol Biol. 2016 Apr 14;16:81. doi: 10.1186/s12862-016-0649-5 (PMC4832473; doi:10.1186/s12862-016-0649-5)
Supplement: Additional file 11: Table S3. — Details of isolates included in the phylogenetic analyses and species delimitation. (DOCX 39 kb) [file 12862_2016_649_MOESM11_ESM.docx]

Table S1 Details of isolates included in the phylogenetic analyses and species delimitation.

| **Species** | **Accession number (h)** |  |  | **GenBank accessions** | | | | | | | |
| --- | --- | --- | --- | --- | --- | --- | --- | --- | --- | --- | --- |
|  |  | **Host** | **Locality** | **ApMat** | **Apn25L** | **CAL** | **GAPDH** | **GS** | **ITS** | **MAT1-2-1** | **TUB2** |
| *C. aenigma* | ICMP 18608* (h) | *Persea americana* | Israel | KM360143 | \ | JX009683 | JX010044 | JX010078 | JX010244 | \ | JX010389 |
| *C. alatae* | ICMP 17919, CBS 304.67* (h) | *Dioscorea alata* | India | KC888932 | \ | JX009738 | JX009990 | JX010065 | JX010190 | \ | JX010383 |
| *C. aotearoa* | ICMP 18537* (h) | *Coprosma* sp. | New Zealand | KC888930 | \ | JX009611 | JX010005 | JX010113 | JX010205 | \ | JX010420 |
| *C. fructicola* | LC3430, LF652 (h) | *Camellia sinensis* | China, Jiangxi | KJ954598 | \ | KJ954743 | KJ954893 | KJ955042 | KJ955192 | \ | KJ955339 |
|  | LC3462, LF686 (h) | *Camellia sinensis* | China, Jiangxi | KJ954606 | \ | KJ954751 | KJ954901 | KJ955050 | KJ955200 | \ | KJ955347 |
|  | ICMP 18646 (h) | *Tetragastris panamensis* | Panama | \ | \ | JX009674 | JX010032 | JX010099 | JX010173 | \ | JX010409 |
|  | ICMP 18581, CBS 130416, MFU 090228* (h) | *Coffea arabica* | Thailand | JQ807838 | \ | FJ917508 | JX010033 | JX010095 | JX010165 | \ | JX010405 |
| *C. gloeosporioides* | IMI 356878, CBS 112999, ICMP 17821* (h) | *Citrus sinensis* | Italy | JQ807843 | \ | JX009731 | JX010056 | JX010085 | JX010152 | \ | JX010445 |
|  | LC3312, LF534 (h) | *Camellia sinensis* | China, Jiangxi | KJ954569 | \ | KJ954710 | KJ954859 | KJ955009 | KJ955158 | \ | KJ955305 |
| *C. henanense* | LC2821, LF25 (h) | *Cirsium japonicum* | China, Beijing | KM610175 | \ | KM610177 | KM610179 | KM610181 | KM610183 | \ | KM610185 |
|  | LC2820, LF24 (h) | *Cirsium japonicum* | China, Beijing | KM610174 | \ | KM610176 | KM610178 | KM610180 | KM610182 | \ | KM610184 |
|  | LC3030, LF238, CGMCC 3.17354* (h) | *Camellia sinensis* | China, Henan | KJ954524 | \ | KJ954662 | KJ954810 | KJ954960 | KJ955109 | \ | KJ955257 |
| *C. horii* | ICMP 10492, NBRC 7478* (h) | *Diospyros kaki* | Japan | JQ807840 | \ | JX009604 | GQ329681 | JX010137 | GQ329690 | \ | JX010450 |
| *C. kahawae subsp. ciggaro* | ICMP 18534* (h) | *Kunzea ericoides* | New Zealand | HE655657 | \ | JX009634 | JX009904 | JX010116 | JX010227 | \ | JX010427 |
| *C. kahawae subsp. kahawae* | IMI 319418, ICMP 17816* (h) | *Coffea arabica* | Kenya | JQ894579 | \ | JX009642 | JX010012 | JX010130 | JX010231 | \ | JX010444 |
| *C. queenslandicum* | CPC 17123 (h) | *Syzygium australa* | Australia | **KP703778** | **KP703197** | **KP703611** | **KP703282** | **KP703693** | **KP703357** | **KP703526** | **KP703439** |
|  | ICMP 1778* (h) | *Carica papaya* | Australia | KC888928 | \ | JX009691 | JX009934 | JX010104 | JX010276 | \ | JX010414 |
| *C. siamense* | LC0144, PE004-1 (h) | *Coffea* sp. | China, Yunnan | **KP703785** | **KP703204** | **KP703618** | **KP703289** | **KP703700** | **KP703391** | **KP703533** | **KP703446** |
|  | LC0147, PE006-1 (h) | *Coffea* sp. | China, Yunnan | **KP703786** | **KP703205** | **KP703619** | **KP703290** | **KP703701** | **KP703362** | **KP703534** | **KP703447** |
|  | LC0148, PE007-1 (h) | *Camellia* sp. | China, Yunnan | KJ954494 | **KP703206** | KJ954631 | KJ954779 | KJ954929 | KJ955078 | **KP703535** | KJ955227 |
|  | LC0149, PE007-2 (h) | *Camellia* sp. | China, Yunnan | KJ954495 | KP703207 | KJ954632 | KJ954780 | KJ954930 | KJ955079 | **KP703536** | KJ955228 |
|  | LC1387, MFLUCC11-0326, KSU-A3 (h) | unknown | Turkey | **KP703787** | **KP703208** | **KP703621** | **KP703292** | **KP703703** | **KP703421** | \ | **KP703449** |
|  | LC1512, MFLUCC11-0327, KSU-A4 | *Persea americana* | Saudi Arabia | **KP703788** | **KP703209** | **KP703622** | **KP703293** | **KP703704** | **KP703416** | **KP703538** | **KP703450** |
|  | LC1518, MFLUCC11-0325, KSU-A2 | *Persea americana* | Saudi Arabia | **KP703789** | **KP703210** | **KP703623** | **KP703294** | **KP703705** | **KP703422** | \ | KP703451 |
|  | LC2838, LF42 (h) | unknown | China, Yunnan | **KP703803** | **KP703228** | **KP703637** | **KP703308** | **KP703719** | **KP703371** | **KP703556** | **KP703465** |
|  | LC2860, LF66 (h) | unknown | China, Yunnan | **KP703805** | **KP703230** | **KP703638** | **KP703309** | **KP703720** | **KP703427** | **KP703558** | **KP703466** |
|  | LC2875, LF81 (h) | unknown | China, Yunnan | **KP703829** | **KP703254** | **KP703662** | **KP703333** | **KP703744** | **KP703394** | **KP703582** | **KP703490** |
|  | LC2876, LF82 | unknown | China, Yunnan | **KP703830** | **KP703255** | **KP703663** | **KP703334** | **KP703745** | **KP703389** | **KP703583** | **KP703491** |
|  | LC2877, LF83 (h) | unknown | China, Yunnan | **KP703831** | **KP703256** | **KP703664** | **KP703335** | **KP703746** | **KP703392** | **KP703584** | **KP703492** |
|  | LC2878, LF84 (h) | unknown | China, Yunnan | **KP703832** | **KP703257** | **KP703665** | **KP703336** | **KP703747** | **KP703386** | **KP703585** | **KP703493** |
|  | LC2931, LF139 (h) | *Camellia* sp. | China, Sichuan | KJ954503 | **KP703211** | KJ954640 | KJ954788 | KJ954938 | KJ955087 | **KP703539** | KJ955236 |
|  | LC2937, LF145 | *Cleyera japonica* | China, Sichuan | **KP703790** | **KP703212** | **KP703624** | **KP703295** | **KP703706** | **KP703363** | **KP703540** | **KP703452** |
|  | LC2939, LF147 (h) | *Cleyera japonica* | China, Sichuan | **KP703791** | **KP703213** | **KP703625** | **KP703296** | **KP703707** | **KP703364** | **KP703541** | **KP703453** |
|  | LC2940, LF148 (h) | *Camellia* sp. | China, Sichuan | KJ954504 | **KP703214** | KJ954641 | KJ954789 | KJ954939 | KJ955088 | **KP703542** | KJ955237 |
|  | LC2941, LF149 (h) | *Camellia* sp. | China, Sichuan | KJ954505 | **KP703215** | KJ954642 | KJ954790 | KJ954940 | KJ955089 | **KP703543** | KJ955238 |
|  | LC2946, LF154 (h) | *Cleyera japonica* | China, Sichuan | **KP703792** | **KP703216** | **KP703626** | **KP703297** | **KP703708** | **KP703366** | **KP703544** | **KP703454** |
|  | LC2957, LF165 (h) | *Phillyrea* sp. | China, Sichuan | **KP703793** | **KP703217** | **KP703627** | **KP703298** | **KP703709** | **KP703372** | **KP703545** | **KP703455** |
|  | LC2963, LF171 (h) | *Schima superba* | China, Jiangxi | **KP703794** | **KP703218** | **KP703628** | **KP703299** | **KP703710** | **KP703423** | **KP703546** | **KP703456** |
|  | LC2964, LF172 (h) | *Schima superba* | China, Jiangxi | **KP703795** | **KP703219** | **KP703629** | **KP703300** | **KP703711** | **KP703367** | **KP703547** | **KP703457** |
|  | LC2965, LF173 (h) | *Schima superba* | China, Jiangxi | **KP703796** | **KP703220** | **KP703630** | **KP703301** | **KP703712** | **KP703408** | **KP703548** | **KP703458** |
|  | LC2966, LF174 (h) | *Schima superba* | China, Jiangxi | **KP703797** | **KP703221** | **KP703631** | **KP703302** | **KP703713** | **KP703365** | **KP703549** | **KP703459** |
|  | LC2967, LF175 (h) | *Schima superba* | China, Jiangxi | **KP703798** | **KP703222** | **KP703632** | **KP703303** | **KP703714** | **KP703373** | **KP703550** | **KP703460** |
|  | LC2968, LF176 (h) | *Schima superba* | China, Jiangxi | **KP703799** | **KP703223** | **KP703633** | **KP703304** | **KP703715** | **KP703374** | **KP703551** | **KP703461** |
|  | LC2969, LF177 (h) | *Camellia oleifera* | China, Jiangxi | KJ954508 | **KP703224** | KJ954645 | \ | KJ954943 | KJ955092 | **KP703552** | KJ955241 |
|  | LC3049, LF257 (h) | *Pittosporum illicioides* | China, Fujian | **KP703800** | **KP703225** | **KP703634** | **KP703305** | **KP703716** | **KP703395** | **KP703553** | **KP703462** |
|  | LC3050, LF258 (h) | *Pittosporum illicioides* | China, Fujian | **KP703801** | **KP703226** | **KP703635** | **KP703306** | **KP703717** | **KP703369** | **KP703554** | **KP703463** |
|  | LC3051, LF259 (h) | *Pittosporum illicioides* | China, Fujian | **KP703802** | **KP703227** | **KP703636** | **KP703307** | **KP703718** | **KP703400** | **KP703555** | **KP703464** |
|  | LC3409, LF631 (h) | *Camellia sinensis* | China, Jiangxi | **KP703804** | **KP703229** | KJ954739 | KJ954888 | KJ955037 | KJ955187 | **KP703557** | KJ955334 |
|  | LC3509, LF737 (h) | unknown | China, Jiangxi | **KP703806** | **KP703231** | **KP703639** | **KP703310** | **KP703721** | **KP703376** | **KP703559** | **KP703467** |
|  | LC3520, LF748 (h) | *Prunus* sp. | China, Jiangxi | **KP703807** | **KP703232** | **KP703640** | **KP703311** | **KP703722** | **KP703401** | **KP703560** | **KP703468** |
|  | LC3521, LF749 (h) | unknown | China, Jiangxi | **KP703808** | **KP703233** | **KP703641** | **KP703312** | **KP703723** | **KP703402** | **KP703561** | **KP703469** |
|  | LC3522, LF750 (h) | unknown | China, Jiangxi | **KP703809** | **KP703234** | **KP703642** | **KP703313** | **KP703724** | **KP703425** | **KP703562** | **KP703470** |
|  | LC3524, LF752 (h) | *Cinnamomum camphora* | China, Jiangxi | **KP703810** | **KP703235** | **KP703643** | **KP703314** | **KP703725** | **KP703384** | **KP703563** | **KP703471** |
|  | LC3526, LF754 (h) | unknown | China, Jiangxi | **KP703811** | **KP703236** | **KP703644** | **KP703315** | **KP703726** | **KP703399** | **KP703564** | **KP703472** |
|  | LC3527, LF755 | unknown | China, Jiangxi | **KP703812** | **KP703237** | **KP703645** | **KP703316** | **KP703727** | **KP703403** | **KP703565** | **KP703473** |
|  | LC3528, LF756 | unknown | China, Jiangxi | **KP703813** | **KP703238** | **KP703646** | **KP703317** | **KP703728** | **KP703404** | **KP703566** | **KP703474** |
|  | LC3530, LF758 (h) | unknown | China, Jiangxi | **KP703814** | **KP703239** | **KP703647** | **KP703318** | **KP703729** | **KP703385** | **KP703567** | **KP703475** |
|  | LC3532, LF760 (h) | unknown | China, Jiangxi | **KP703815** | **KP703240** | **KP703648** | **KP703319** | **KP703730** | **KP703407** | **KP703568** | **KP703476** |
|  | LC3533, LF761 (h) | unknown | China, Jiangxi | **KP703816** | **KP703241** | **KP703649** | **KP703320** | **KP703731** | **KP703426** | **KP703569** | **KP703477** |
|  | LC3534, LF762 | unknown | China, Jiangxi | **KP703817** | **KP703242** | **KP703650** | **KP703321** | **KP703732** | **KP703398** | **KP703570** | **KP703478** |
|  | LC3536, LF764 (h) | unknown | China, Jiangxi | **KP703818** | **KP703243** | **KP703651** | **KP703322** | **KP703733** | **KP703368** | **KP703571** | **KP703479** |
|  | LC3538, LF766 (h) | unknown | China, Jiangxi | **KP703819** | **KP703244** | **KP703652** | **KP703323** | **KP703734** | **KP703396** | **KP703572** | **KP703480** |
|  | LC3540, LF768 (h) | unknown | China, Jiangxi | **KP703820** | **KP703245** | **KP703653** | **KP703324** | **KP703735** | **KP703406** | **KP703573** | **KP703481** |
|  | LC3542, LF770 (h) | unknown | China, Jiangxi | **KP703821** | **KP703246** | **KP703654** | **KP703325** | **KP703736** | **KP703405** | **KP703574** | **KP703482** |
|  | LC3543, LF771 (h) | unknown | China, Jiangxi | **KP703822** | **KP703247** | **KP703655** | **KP703326** | **KP703737** | **KP703397** | **KP703575** | **KP703483** |
|  | LC3544, LF772 (h) | unknown | China, Jiangxi | **KP703823** | **KP703248** | **KP703656** | **KP703327** | **KP703738** | **KP703409** | **KP703576** | **KP703484** |
|  | LC3549, LF777 (h) | *Ilex chinensis* | China, Hangzhou | **KP703824** | **KP703249** | **KP703657** | **KP703328** | **KP703739** | **KP703393** | **KP703577** | **KP703485** |
|  | LC3550, LF778 (h) | *Ilex chinensis* | China, Hangzhou | **KP703825** | **KP703250** | **KP703658** | **KP703329** | **KP703740** | **KP703383** | **KP703578** | **KP703486** |
|  | LC3551, LF779 | *Ilex chinensis* | China, Hangzhou | **KP703826** | **KP703251** | **KP703659** | **KP703330** | **KP703741** | **KP703377** | **KP703579** | **KP703487** |
|  | LC3552, LF780 (h) | *Ilex chinensis* | China, Hangzhou | **KP703827** | **KP703252** | **KP703660** | **KP703331** | **KP703742** | **KP703390** | **KP703580** | **KP703488** |
|  | LC3553, LF781 | *Ilex chinensis* | China, Hangzhou | **KP703828** | **KP703253** | **KP703661** | **KP703332** | **KP703743** | **KP703378** | **KP703581** | **KP703489** |
|  | LC3642, LF871 (h) | *Cinnamomum camphora* | China, Jiangxi | **KP703833** | **KP703258** | **KP703666** | **KP703337** | **KP703748** | **KP703379** | **KP703586** | **KP703494** |
|  | LC3658, LF887 (h) | *Cinnamomum camphora* | China, Jiangxi | **KP703834** | **KP703259** | **KP703667** | **KP703338** | **KP703749** | **KP703380** | **KP703587** | **KP703495** |
|  | LC3662, LF891 (h) | *Cinnamomum camphora* | China, Jiangxi | **KP703835** | **KP703260** | **KP703668** | **KP703339** | **KP703750** | **KP703381** | \ | **KP703496** |
|  | LC3663, LF892 (h) | *Cinnamomum camphora* | China, Jiangxi | **KP703836** | **KP703261** | **KP703669** | **KP703340** | **KP703751** | **KP703382** | **KP703588** | **KP703497** |
|  | LC3672, LF902 (h) | *Cinnamomum* sp. | China, Jiangxi | **KP703837** | **KP703262** | **KP703670** | **KP703341** | **KP703752** | **KP703375** | **KP703589** | **KP703498** |
|  | LC3682, LF912 (h) | *Schima superba* | China, Jiangxi | **KP703839** | **KP703264** | **KP703672** | **KP703343** | **KP703754** | **KP703387** | **KP703591** | **KP703500** |
|  | LC3684, LF914 (h) | *Osmanthus* sp. | China, Jiangxi | **KP703840** | **KP703265** | **KP703673** | **KP703344** | **KP703755** | **KP703388** | **KP703592** | **KP703501** |
|  | CBS 440.67 (h) | *Coffea* sp. | Kenya; Ruiru | **KP703772** | **KP703191** | **KP703605** | **KP703276** | **KP703686** | **KP703356** | **KP703522** | **KP703433** |
|  | CBS 129.91 (h) | *Azadirachta indica* |  | **KP703768** | **KP703184** | **KP703602** | **KP703273** | **KP703683** | **KP703350** | **KP703515** | **KP703432** |
|  | CBS 116868 (h) | *Musa* sp. | India; Southern India | **KP703764** | **KP703179** | **KP703599** | KC566669 | **KP703679** | KC566815 | **KP703510** | **KP703429** |
|  | CBS 116869 | *Musa* sp. | India; Southern India | **KP703765** | **KP703180** | **KP703600** | KC566670 | **KP703680** | KC566816 | **KP703511** | **KP703430** |
|  | CPC 16135, WTS9 | *Persea americana* | South Africa | **KP703845** | **KP703270** | **KP703678** | **KP703349** | **KP703760** | **KP703414** | **KP703597** | **KP703506** |
|  | CPC 16136, WTS10 | *Persea americana* | South Africa | **KP703843** | **KP703268** | **KP703676** | **KP703347** | **KP703758** | **KP703417** | **KP703595** | **KP703504** |
|  | CPC 16137, WTS11 | *Persea americana* | South Africa | **KP703844** | **KP703269** | **KP703677** | **KP703348** | **KP703759** | **KP703418** | **KP703596** | **KP703505** |
|  | IMI 96858, CPC 16807 (h) | *Annona squamosa* | India, Allahabad | **KP703784** | **KP703203** | **KP703617** | **KP703288** | **KP703699** | **KP703361** | **KP703532** | **KP703445** |
|  | CBS 125969, NB 865 (h) | Cactus | Turkey | **KP703842** | **KP703267** | **KP703675** | **KP703346** | **KP703757** | **KP703352** | **KP703594** | **KP703503** |
|  | CBS 125971, NB 823 (h) | Dipladenia | Netherlands | **KP703841** | **KP703266** | **KP703674** | **KP703345** | **KP703756** | **KP703410** | **KP703593** | **KP703502** |
|  | CPC 15983 (h) | *Musa* sp. | Mexico | **KP703777** | **KP703196** | **KP703610** | **KP703281** | **KP703692** | **KP703351** | **KP703525** | **KP703438** |
|  | CPC 14473 (h) | *Persea americana* |  | **KP703773** | **KP703192** | **KP703606** | **KP703277** | **KP703688** | **KP703412** | **\** | **KP703434** |
|  | CPC 14474 (h) | *Persea americana* |  | **KP703774** | **KP703193** | **KP703607** | **KP703277** | **KP703689** | **KP703415** | **KP703523** | **KP703435** |
|  | CPC 14475 | *Persea americana* |  | **KP703775** | **KP703194** | **KP703608** | **KP703277** | **KP703690** | **KP703413** | **\** | **KP703436** |
|  | IMI 351902, CPC 18922 (h) | *Bombax malabaricum* | India | **KP703782** | **KP703201** | **KP703615** | **KP703286** | **KP703697** | **KP703358** | **KP703530** | **KP703443** |
|  | CBS 125470 | *Coffea* sp. | Vietnam;Yen Bai | **KP703767** | **KP703183** | **KP703601** | **KP703272** | **KP703682** | **KP703419** | **KP703514** | **\** |
|  | CBS 130417, ICMP 18578, LC0034, BPD-I 2* (h) | *Coffea arabica* | Thailand | **KP703769** | **KP703185** | JX009714 | JX009924 | JX010094 | JX010171 | **KP703516** | JX010404 |
|  | CPC 18851 (h) | unknown | Brazil | **KP703781** | **KP703200** | **KP703614** | **KP703285** | **KP703696** | **KP703354** | **KP703529** | **KP703442** |
|  | CBS 113199. CPC 2290 | *Protea cynaroides* | Zimbabwe | **KP703763** | **KP703178** | KC296962 | KC297008 | KC297031 | KC297066 | **KP703509** | KC297090 |
|  | CBS 112985, IMI 319424, CPC 5295 (h) | *Coffea arabica* | Kenya | **KP703762** | **KP703177** | **KP703598** | **KP703271** | **KP703687** | **KP703411** | **KP703508** | **KP703428** |
|  | CBS 112983, CPC 2291 (h) | *Protea cynaroides* | Zimbabwe | **KP703761** | **KP703176** | KC296961 | KC297007 | KC297030 | KC297065 | **KP703507** | KC297100 |
|  | IMI 82267, CPC 16808 (h) | *Vitis* sp. | Brazil | **KP703783** | **KP703202** | **KP703616** | **KP703287** | **KP703698** | **KP703355** | **KP703531** | **KP703444** |
|  | CPC 18478 (h) | unknown | Brazil | **KP703780** | **KP703199** | **KP703613** | **KP703284** | **KP703695** | **KP703420** | **KP703528** | **KP703441** |
|  | CPC 18452 (h) | unidentified berry plant | Brazil | **KP703779** | **KP703198** | **KP703612** | **KP703283** | **KP703694** | **KP703359** | **KP703527** | **KP703440** |
| *C. siamense* (syn. *C. communis*) | NK24, MTCC 11599* | *Mangifera indica* | India | JQ894582 | \ | KC790791 | JQ894632 | \ | JQ894681 | \ | JQ894602 |
| *C. siamense* (syn. *C. dianesei*) | CMM 4083, MFLU 1300058* | *Mangifera indica* | Brazil | KJ155461 | \ | KC517209 | KC517194 | KC430894 | KC329779 | \ | KC517254 |
|  | CMM 4082, MFLU 1300057 | *Mangifera indica* | Brazil | KJ155460 | \ | KC517204 | KC517157 | KC430895 | KC329774 | \ | KC517249 |
|  | CMM 4084 | *Mangifera indica* | Brazil | KJ155462 | \ | KC517228 | KC517201 | KC430890 | KC329811 | \ | KC517273 |
|  | CMM 4085 | *Mangifera indica* | Brazil | KJ155463 | \ | KC517230 | KC517196 | KC430891 | KC329813 | \ | KC517275 |
|  | CMM 3740 | *Mangifera indica* | Brazil | KJ155452 | \ | KC992371 | KC702954 | \ | KC702978 | \ | KC702921 |
| *C. siamense* (syn. *C. endomangiferae*) | CMM 3814* | *Mangifera indica* | Brazil | KJ155453 | \ | KC992372 | KC702955 | \ | KC702994 | \ | KC702922 |
| *C. siamense* (syn. *C. hymenocallidis*) | CBS 125378, ICMP 18642, LC0043, CSSN2* (h) | *Hymenocallis americana* | China | JQ899283 | **KP703182** | JX009709 | JX010019 | JX010100 | JX010278 | **KP703513** | JX010410 |
| *C. siamense* (syn. *C. jasmini-sambac*) | CBS 130420, ICMP 19118, LC0921, LLTA-01* (h) | *Jasminum sambac* | Vietnam | JQ899273 | **KP703186** | JX009713 | HM131497 | JX010105 | HM131511 | **KP703517** | JX010415 |
|  | CBS 133123, coll126 | *Vaccinium macrocarpon* | USA, New Jersey | JX145309 | **KP703187** | **KP703603** | **KP703274** | **KP703684** | JX145142 | **KP703518** | JX145193 |
| *C. siamense* (syn. *C. melanocaulon*) | CBS 133251, coll131, BPI 884113* (h) | *Vaccinium macrocarpon* | USA, New Jersey | JX145313 | **KP703190** | **KP703604** | **KP703275** | **KP703685** | JX145144 | **KP703521** | JX145195 |
| *C. siamense* (syn. *C. murrayae* (illeg. nom.)) | CBS 133239, GZAAS5.09506* (h) | *Murraya* sp. | China | **KP703770** | **KP703188** | JQ247596 | JQ247609 | JQ247621 | JQ247633 | **KP703519** | JQ247644 |
|  | CBS 133240, GZAAS5.09538 (h) | *Murraya* sp. | China | **KP703771** | **KP703189** | JQ247597 | JQ247608 | JQ247620 | JQ247632 | **KP703520** | JQ247645 |
| *C. theobromicola* | CBS 124945, ICMP 18649* (h) | *Theobroma cacao* | Panama | KC790726 | \ | JX009591 | JX010006 | JX010139 | JX010294 | \ | JX010447 |
| *C. tropicale* | CBS 124943, GJS 08-42 (h) | *Annona muricata* | Panama | **KP703766** | **KP703181** | JX009720 | JX010014 | **KP703681** | **KP703360** | **KP703512** | **KP703431** |
|  | CBS 124949, ICMP 18653* (h) | *Theobroma cacao* | Panama | KC790728 | \ | JX009719 | JX010007 | JX010097 | JX010264 | \ | JX010407 |
| *C. xanthorrhoeae* | ICMP 17903, CBS 127831, BRIP 45094* (h) | *Xanthorrhoea preissii* | Australia | KC790689 | \ | JX009653 | JX009927 | JX010138 | JX010261 | \ | JX010448 |

Note: (h): The unique haplotypes used in the GMYC, PTP and BP&P analyses; GenBank accessions numbers in bold were newly generated in this study.
