# Supplementary material for: Species boundaries in plant pathogenic fungi: a Colletotrichum case study
Source: BMC Evol Biol. 2016 Apr 14;16:81. doi: 10.1186/s12862-016-0649-5 (PMC4832473; doi:10.1186/s12862-016-0649-5)
Supplement: Additional file 12: Table S4 — Primers used in this study, with sequences and sources. (DOCX 14 kb) [file 12862_2016_649_MOESM12_ESM.docx]

Table S2 Primers used in this study, with sequences and sources.

| Locus | Primer name | Direction | Sequence (5'-3') | Reference |
| --- | --- | --- | --- | --- |
| ApMat | AMF1 | Forward | CCAGAAATACACCGAACTTGC | Silva et al. 2012 |
|  | AMR1 | Reverse | TCATTCTACGTATGTGCCCG | Silva et al. 2012 |
| Apn25L | A5L-F | Forward | CAAGCGACGAAGTATACGAG | Silva et al. 2012 |
|  | A5L-R | Reverse | GCATCACGGGAATAACTAGG | Silva et al. 2012 |
| CAL | CL1C | Forward | GAA TTC AAG GAG GCC TTC TC | Weir et al. 2012 |
|  | CL2C | Reverse | CTT CTG CAT CAT GAG CTG GAC | Weir et al. 2012 |
| GAPDH | GDF1 | Forward | GCCGTCAACGACCCCTTCATTGA | Templeton et al. 1992 |
|  | GDR1 | Reverse | GGGTGGAGTCGTACTTGAGCATGT | Templeton et al. 1992 |
| GS | GSF1 | Forward | ATGGCCGAGTACATCTGG | Stephenson et al. 1997 |
|  | GSLF2 | Forward | TACACGAGSAAAAGGATACGC | This study |
|  | GSLF3 | Forward | GATACGCCTCTTCCAGCGTT | This study |
|  | GSR1 | Reverse | GAACCGTCGAAGTTCCAC | Stephenson et al. 1997 |
|  | GSLR1 | Reverse | AGRCGCACATTGTCAGTATCG | This study |
| ITS | ITS1 | Forward | TCCGTAGGTGAACCTGCGG | White et al. 1990 |
|  | ITS4 | Reverse | TCCTCCGCTTATTGATATGC | White et al. 1990 |
| TUB2 | T1 | Forward | AACATGCGTGAGATTGTAAGT | O'Donnell & Cigelnik 1997 |
|  | Bt2b | Reverse | ACCCTCAGTGTAGTGACCCTTGGC | Glass & Donaldson 1995 |
| MAT1-2-1 | CM-F | Forward | TCTACCTCATCGACGCTGCT | Silva et al. 2012 |
|  | M5L-R | Reverse | GACCCTTCTATGAACGAGCC | Silva et al. 2012 |
